# Early stroke detection through machine learning in the prehospital setting

**DOI:** 10.3389/fcvm.2025.1629853

**Published:** 2025-08-07

**Authors:** María Ríos Delgado, Gemma Reig Roselló, Nicolas Riera-Lopez, José A. Vivancos, José L. Ayala

**Affiliations:** ^1^ Department of Computer Arquitecture and Automation, Universidad Complutense de Madrid, Madrid, Spain; ^2^ Servicio de Neurología, Hospital Universitario de La Princesa, IIS-Princesa – Instituto de Investigación Sanitaria Hospital Universitario de La Princesa, Madrid, Spain; ^3^ Stroke Commission, Madrid Emergency Medical Service (SUMMA 112), Madrid, Spain

**Keywords:** stroke, LVO, emergency medical services, prehospital, machine learning, genetic algorithms, clinical data, hemodynamic data

## Abstract

**Background:**

Stroke is a leading cause of death and disability globally, with rising prevalence driven by modern lifestyle factors. Despite the critical nature of stroke as a time-sensitive condition requiring prompt diagnosis and intervention, current pre-diagnostic practices are often limited by reliance on specific patient symptoms, which can delay appropriate treatment, especially for large vessel occlusions (LVO). This study introduces a novel approach utilizing machine learning techniques to accurately identify stroke type and severity using hemodynamic data. By enhancing the pre-hospital diagnosis process, the research aims to optimize hospital selection and improve emergency stroke care, ultimately ensuring timely treatment at specialized centers.

**Methods:**

The methodology of this project consists on two phases. The first step involves developing two specialized models to predict the type of stroke—ischemic or hemorrhagic—along with a Bayesian rule to determine the final classification. The second step, applied only in cases of ischemic stroke, identifies whether the episode is a Large Vessel Occlusion (LVO) or not.

**Results:**

The study developed a robust framework for detecting Large Vessel Occlusions (LVO) during Emergency Medical Services (EMS) interventions. The results for ischemic episodes showed that the LVO model achieved 91.67% recall and 64.71% precision, outperforming the prehospital scale used as a reference in all performance metrics except specificity. This model utilized only 20 out of the 271 original variables, with the most representative variables including blood pressure, heart rate, oxygen saturation, and arm movement. The integration of the LVO model for the complete sample with a Bayesian pipeline resulted in a precision of 59% and a recall of 74%, while applying the LVO model to the entire population yielded a precision of 60.60% and a recall of 80.19%.

**Conclusion:**

The study concluded that the implementation of Machine Learning (ML) techniques can significantly improve the diagnostic accuracy of stroke in the context of Emergency Medical Services (EMS). The LVO model demonstrated promising results, with an improvement in positive recall of approximately 10%–13% compared to the baseline paradigm. The use of objective variables, such as blood pressure and heart rate, was a key factor in this enhancement. The study highlights the potential benefits of leveraging ML techniques in Emergency Medicine, particularly in the diagnosis and management of stroke. The results suggest that the LVO model can potentially augment the precision of stroke diagnosis, facilitating more efficacious and timely interventions.

## 1 Introduction

Stroke is one of the most prevalent and devastating medical conditions worldwide, with one in four individuals expected to experience a stroke during their lifetime (1). The prevalence of stroke is increasing, fueled by modern lifestyle factors such as sedentary behavior, unhealthy diets, and aging populations. This growing public health concern underscores the urgent need for advancements in diagnostic and treatment approaches. Stroke remains the leading cause of death among women and a major contributor to adult disability globally. Its consequences range from significant physical and cognitive impairments to a considerable reduction in quality of life or even death. Given its profound impact, stroke is often regarded as a critical time-sensitive medical emergency where prompt diagnosis and treatment are paramount for ensuring favorable outcomes (2).

Early and accurate diagnosis of stroke plays a pivotal role in determining the course of treatment and improving the patient’s prognosis. Broadly, strokes are categorized into ischemic, caused by the obstruction of blood flow to the brain due to clots, and hemorrhagic, resulting from blood vessel rupture. Among ischemic strokes, large vessel occlusions (LVO) are particularly severe due to their potential to compromise larger areas of brain function. Effective management of these conditions hinges on specialized treatment pathways: hemorrhagic strokes often require blood pressure control, minor ischemic strokes may benefit from fibrinolytic therapy, and LVO cases typically demand mechanical thrombectomy. However, not all healthcare facilities are equipped to provide thrombectomy or advanced neuroimaging capabilities, creating a pressing need for prehospital tools that can triage and prioritize patients effectively.

In Spain, the implementation of the *stroke code* system addresses these challenges by employing prehospital scales, such as the Madrid Direct scale (3, 4), to assess the need for thrombectomy during emergency response. However, these tools rely heavily on observable clinical symptoms, which may lead to under- or over-triage in cases of LVO (5), delaying timely intervention and necessitating secondary transfers. Such limitations highlight the potential of machine learning (ML) to revolutionize prehospital stroke care.

The application of Machine Learning (ML) in stroke medicine offers immense promise, with algorithms capable of analyzing complex datasets—ranging from neuroimaging and clinical parameters to wearable and hemodynamic data. ML has already demonstrated its utility in stroke research (6), from improving diagnostic precision to predicting outcomes and optimizing treatment plans. Its ability to uncover latent patterns in large datasets and make real-time predictions can enhance decision-making and streamline workflows in emergency medical services (EMS). Recent studies have explored the use of ML for stroke diagnosis, including the analysis of biosignals such as ECG. Ensemble models have shown strong performance in detecting cardiac abnormalities from ECG data (7) LSTM networks have been successfully combined with clinical parameters to predict stroke-related conditions (8). Additionally, ML models using hemodynamic data have shown potential for automatic stroke classification in intensive care settings (9). However, most of these studies are limited to in-hospital environments and are not designed for real-time application in the field.

Recent efforts have explored the feasibility of ML-based stroke diagnosis in prehospital settings. Kummer et al. (10) applied natural language processing (NLP) to paramedic narratives to detect general stroke presence during EMS encounters. While their model improved recognition rates, it did not distinguish between stroke subtypes or identify large vessel occlusion (LVO), a critical factor in triage for thrombectomy. Ong et al. (11) combined ML and NLP to analyze radiology reports, achieving good performance in classifying ischemic stroke and determining lesion location. However, this method is reliant on imaging reports and thus not applicable to the EMS phase, where real-time, sensor-based data is essential.

Other studies have trained deep learning models on ECG signals to detect stroke (12) and demonstrated the use of NLP techniques to identify stroke patients from emergency department triage notes (13). ML has also been used to predict stroke-related outcomes, including the identification of key prognostic factors from electronic health records (EHRs) (14) and the estimation of stroke onset time from imaging data (15, 16). These studies illustrate the versatility of ML in stroke care, yet most remain anchored in hospital workflows and lack direct application to emergency transport contexts.

The application of ML in prehospital stroke care is promising, but it also presents unique challenges such as limited access to data, computational resources, and reliable network connections in ambulances. Integrating ML into clinical workflows requires robust preprocessing, real-time computation, and seamless integration with existing protocols, while hemodynamic data collected during patient transport introduces complexities like signal noise and variability.

Despite these challenges, ML can provide accurate and timely diagnosis, reducing the time from stroke onset to treatment, improving patient outcomes, and decreasing the economic burden on healthcare systems. A ML-based framework combining clinical and hemodynamic data can support automated triage and optimized hospital routing, potentially revolutionizing prehospital stroke care.

This work leverages ML algorithms to classify stroke type (ischemic vs. hemorrhagic) and identify severity (LVO vs. non-LVO) using hemodynamic and clinical data gathered during EMS transport. By integrating ML with genetic algorithms for variable selection, the study aims to create a more objective and accurate pre-diagnostic tool, enabling EMS professionals to make informed transport and treatment decisions. The interdisciplinary nature of this approach, blending clinical expertise with cutting-edge ML techniques, underscores its potential to transform stroke management and improve patient outcomes in resource-constrained, prehospital settings.

## 2 Materials and methods

### 2.1 Study design and included datasets

For this observational study, we prospectively collected data on patients who underwent **prehospital stroke code activation** by SUMMA112 in Madrid throughout 2022. All stroke codes activated by EMS were included in this analysis.

The **Madrid Stroke Code** is activated for patients presenting with suspected acute stroke symptoms. Additionally, patients must exhibit a current neurological deficit at the time of assessment, characterized by at least one of the following symptoms: sudden numbness, weakness, or paralysis of the face, arm, or leg on one side of the body; sudden confusion; difficulty speaking or understanding speech; acute vision loss in one or both eyes; severe, abrupt headache with no apparent cause, often accompanied by nausea and vomiting not attributable to other conditions; or impaired gait, loss of balance, or coordination.

Exclusion criteria for Stroke Code include the following: a time lapse of more than 24 h since the onset of symptoms; a patient with significant pre-existing dependency; a clinical situation characterized by severe and irreversible illness that limits life expectancy; and moderate to severe dementia.

Data collection followed a **structured protocol** in which each patient was monitored using a **Lifepack 15 monitor from Stryker**, pre-installed in EMS ambulances. Patients were connected to the monitor for a continuous 10 min period, during which key **physiological parameters** were recorded. The monitoring data was then automatically transferred from the monitor to a dedicated EMS tablet. Alongside physiological data, EMS physicians completed a comprehensive **clinical profile** for each patient, detailing relevant clinical indicators and initial assessments.

Data transfer was conducted in batch mode, with all information automatically uploaded to a centralized data repository where it was securely stored. The raw physiological data, recorded in the proprietary .pco format, was subsequently converted into multiple XML files, each representing distinct aspects of the patient’s monitored data. This conversion enabled a structured and standardized format for later analysis and facilitated linking the data with additional clinical information.

Once the prehospital data was compiled, follow-up information was gathered from hospital clinical records post-treatment, including details on stroke type, severity, treatment administered, and mortality. Patient confidentiality was rigorously protected, with all identifying information hashed to anonymize personal identifiers in compliance with data protection standards.

**Figure 1:**
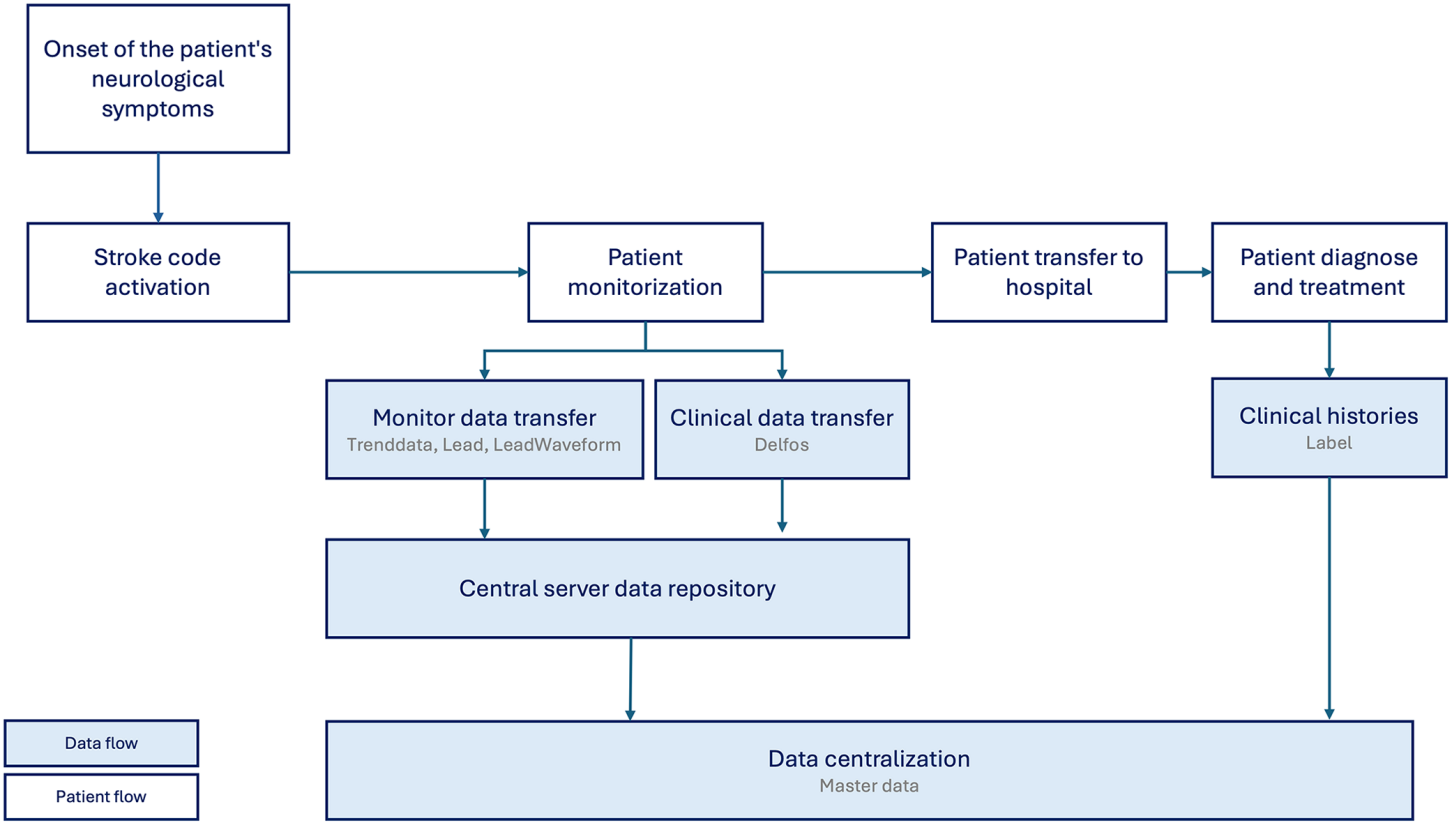
Overview of data collection during stroke code activation. This diagram illustrates the workflow and sources of data acquisition initiated when a stroke code is activated. Data collection begins with prehospital care, including vital signs and initial assessments captured by emergency medical services (EMS). Upon hospital arrival, additional clinical variables, neurological evaluations, and electronic health record (EHR) data are integrated.

The diagnosis and treatment of patients were determined through a thorough review of their clinical histories, which were accessed following hospital admission. This prospective analysis involved examining detailed records of each patient’s symptoms, diagnostic tests, treatments administered, and overall progression of their conditions. Access to these clinical histories allowed for an accurate assessment of each case, enabling the healthcare team to identify patterns, evaluate the effectiveness of treatments, and make informed decisions regarding patient care. In Figure 1 the complete process is described.

The study protocol received approval from the local research ethics committee (22-07-21, acta CEIm 14/21, Registration number 4570).

#### 2.1.1 Clinical data

The clinical dataset, referred to as Delfos, contains 256 variables and encompasses 2,490 stroke code episodes. A comprehensive list of these variables, along with their descriptions and characteristics, is provided in Supplementary Table S1. To summarize, the dataset includes both categorical and numerical variables, with varying levels of completeness.

The Delfos dataset originates from EMS tablets, requiring extensive preprocessing to ensure data quality and consistency. Key steps in this process are outlined below:

1. **Normalization of EMS descriptions.** Fields such as medical history, nursing notes, and treatment descriptions were standardized by removing special characters and converting text to uppercase. Using text extraction techniques, new variables were created, replacing the original ones. Examples include:
  • Specific flags for the administration of drugs (e.g., urapidil, atropine, labetalol, etc.).
  • General flags for drug administration.
  • Flags indicating atrial fibrillation, prior strokes, and anticoagulant use.
2. **Imputation of missing values.** Variables representing clinical scales (e.g., consciousness level, limb strength, and sensitivity) were treated by imputing missing values with *Not Evaluated*.
3. **Conversion to ordinal variables.** Categorical variables with hierarchical relationships were transformed into ordinal scales to better capture their progression. Examples include:
  • Levels of consciousness (*Comatose* to *Conscious*).
  • Orientation metrics (*Time, Person, Place*).
  • Symptom-related variables such as facial paralysis and hemiparesis.
4. **Duplicate handling.** Event duplicates were resolved by concatenating textual variables, retaining the most severe diagnosis for ordinal variables, and selecting maximum values for numerical entries. Patient ID duplicates were excluded if identifiers were missing.
5. **Variable filtering.** Variables with high missingness, redundancy, or irrelevance to modeling objectives were removed.
6. **Minimal missing data strategy.** Variables with minor missing values were left untreated to avoid bias introduction.

The dataset’s overall completeness varies, with notable gaps in specific variables. For instance:

• The Madrid Direct scale features 608 missing values, constituting approximately 24.4% of its entries.
• Incorrect entries account for 5.2% of the dataset.

These figures underscore the critical role of preprocessing.

#### 2.1.2 Hemodynamic monitoring data

The hemodynamic monitoring data is structured in proprietary PCO files, later transformed into XML format. The data spans three primary sources:

• **TrendData.xml:** Continuous measurements recorded every 30 s, including variables such as heart rate, oxygen saturation, blood pressure, and ST-segment metrics.
• **12-Lead N_Waveform.xml:** Ten-second waveforms for each of the 12 ECG leads, stored separately.
• **12-Lead N.xml:** Automatic interpretations and measurements derived from ECG data.

The hemodynamic dataset includes thousands of observations per patient, with vital signs captured at high frequency. However, preprocessing steps were necessary to standardize file formats and resolve inconsistencies. This continuous information has been transformed into static data by obtaining statistical variables such as mean, median, maximum and minimum values, or variation rates.

To ensure analytical continuity, the clinical and hemodynamic datasets were merged by patient IDs and timestamps. This process facilitated the alignment of clinical symptoms with hemodynamic responses, forming the basis for advanced modeling.

Detailed descriptions of the variables in the hemodynamic monitoring data set are provided in Supplementary Table S2. This table includes summaries of variable types, missingness percentages, and examples of recorded values. For example, systolic blood pressure readings range from 80 to 200 mmHg, with a mean of 135 mmHg.

#### 2.1.3 Clinical histories

The clinical histories dataset serves as a crucial component in this study, providing detailed diagnostic and treatment information for each patient. This dataset facilitates the creation of predictive models by supplying labeled data for machine learning algorithms. The dataset consists of 2,177 patient records, each containing key medical details related to stroke diagnosis, large vessel occlusion (LVO), and treatment interventions.The collected variables and their descriptions are presented in Supplementary Table S3.

• **CIPA (Patient ID Hashed):** A unique identifier for each patient, ensuring anonymization and data protection. This variable is stored as a salt hashed string and has no missing values.
• **HOSPITAL (Hospital patient is transferred to):** The name of the hospital where the patient received treatment. This categorical text field provides insights into hospital distribution and potential variations in treatment protocols. A small percentage (0.42%) of values are missing.
• **DATE (Date of incident):** The date on which the stroke event occurred, recorded in a standardized date format. This is a fundamental temporal variable for analyzing trends and time-sensitive aspects of stroke care. No missing values are present.
• **STROKE TYPE (Type of stroke):** A polytomous categorical variable that classifies the stroke as ischemic, hemorrhagic, or other. This is a key variable for distinguishing between different stroke mechanisms and their respective treatments. Approximately 4.09% of values are missing.
• **LVO (Large Vessel Occlusion):** A dichotomous categorical variable indicating whether the ischemic stroke involves a large vessel occlusion (Yes/No). LVO is a critical factor in determining eligibility for mechanical thrombectomy. Missing values account for 4.27%.
• **TROMBOLISIS (Thrombolysis treatment):** A dichotomous variable that indicates whether the patient received thrombolysis (Yes/No). Thrombolysis is a first-line treatment for ischemic stroke within the therapeutic window. Missing values account for 4.27%.
• **THROMBECTOMY (Thrombectomy treatment):** A dichotomous variable specifying whether the patient underwent mechanical thrombectomy (Yes/No). This intervention is primarily used for LVO cases. Missing values account for 4.27%.
• **EXITUS (Death of patient):** A dichotomous variable indicating whether the patient died during hospitalization (Yes/No). This variable provides an essential endpoint for outcome analysis. The highest percentage of missing values (14.88%) is observed in this variable.

This dataset includes information from 2,177 patients, comprising 320 cases of hemorrhagic stroke (14.70%), 1,347 cases of ischemic stroke (61.87%), and 89 cases with missing stroke type data (4.09%). Among ischemic stroke patients, 50.63% were classified as having Large Vessel Occlusion (LVO), a critical parameter in determining emergency treatment strategies.

The structured dataset enables model training and validation, supporting the development of an efficient machine learning-based stroke classification system. By integrating this dataset with hemodynamic monitoring and clinical data, this study aims to enhance early stroke detection and triage in prehospital settings.

### 2.2 Data strategy

**Figure 2:**
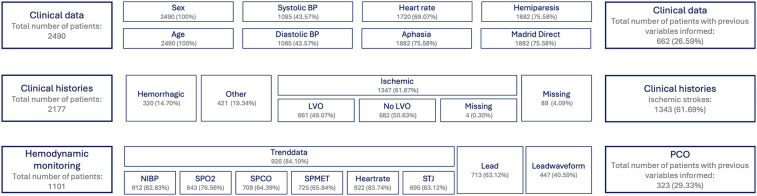
Overview of main datasets and distribution characteristics. This figure summarizes the primary datasets used in the study, including key distributional statistics such as the total number of patients, prevalence of ischemic and hemorrhagic stroke cases, and availability of features across datasets. The visualization highlights differences in sample size, class imbalance, and completeness of variables relevant to predictive modeling.

After preprocessing the three previous datasets, all information is merged into a master dataset. This dataset includes only stroke episodes that are accurately labeled and contain at least one variable from either clinical data or hemodynamic monitoring. In Figure 2, a description of all three sources Final dataset obtained consists of 271 variables and 2,036 observations. No further pre-processing has been applied to the total dataset, meaning missing values are present in the data.

The decision to retain missing values rather than imputing them was made to avoid introducing bias into the results. Imputation techniques, while commonly used, can potentially distort the true distribution of variables, leading to unreliable conclusions in machine learning models. Instead, this study employs techniques that can manage missing values effectively during the modeling phase.

To address missing data, two main strategies are considered:

• **Handling missing data within models:** Some machine learning algorithms, such as decision trees and gradient boosting methods, can handle missing values natively. Additionally, ensemble techniques can incorporate missingness as an informative feature.
• **Eliminating incomplete instances:** In cases where missing data significantly impacts a variable’s integrity, affected observations may be removed from the dataset. This is particularly relevant when a large proportion of missing values exists for critical variables, where imputation is not feasible without bias introduction.

Maintaining missing values requires careful consideration during model training and evaluation. The presence of incomplete data may affect model generalization, necessitating robust validation strategies. Future work may explore advanced techniques such as multiple imputation, missing-indicator methods, or probabilistic modeling to refine the approach to handling missing data in stroke prediction models.

### 2.3 Modeling strategy

**Figure 3:**
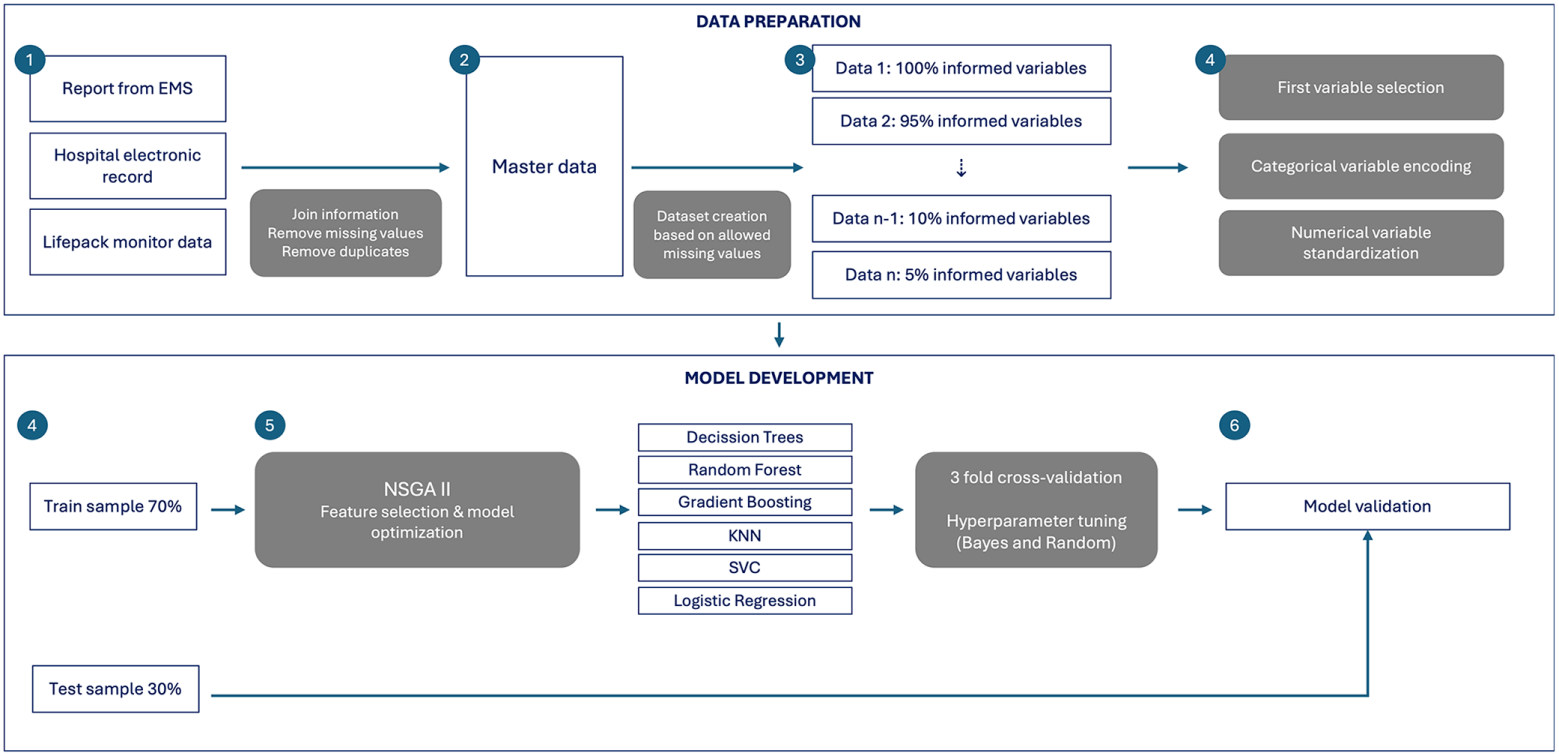
Methodological workflow for feature selection and predictive modeling. The diagram outlines the methodological pipeline used to develop predictive models for stroke subtype classification and severity.

The primary objective of this study is to predict the type of stroke and determine whether it involves a large vessel occlusion (LVO). To achieve this goal, we developed a structured and systematic workflow using custom Python scripts. These scripts are designed to be adaptable, facilitating the creation and refinement of each predictive model in an efficient and reproducible manner. In Figure 3, this model strategy is described.

#### 2.3.1 Workflow overview

The modeling process begins with the construction of a **master dataset**, integrating preprocessed clinical and hemodynamic data. The initial step involves **selecting observations with complete data**. Given the extensive number of variables and the presence of missing values, an iterative filtering process is applied. This method generates multiple datasets, each containing different levels of completeness, allowing for a flexible approach to data inclusion. The process operates in a loop:

• The first iteration includes only cases where all variables are fully available, ensuring high data quality but reducing the number of observations.
• Subsequent iterations progressively relax completeness constraints, allowing a higher number of observations at the expense of additional missing values, down to a threshold of 5% completeness.

This iterative strategy balances the trade-off between data richness and dataset size, ensuring that models are trained on informative but sufficiently large samples.

#### 2.3.2 Preprocessing pipeline

For each generated dataset, a **preprocessing pipeline** is implemented to ensure consistency and enhance model performance. The preprocessing steps include:

• **Variable selection:** Removal of identifiers (e.g., patient IDs, timestamps) that do not contribute to model learning.
• **Encoding categorical variables:** Transformation of categorical data into numerical formats suitable for machine learning algorithms.
• **Standardization of numerical variables:** Normalization of continuous features to ensure uniform scaling across input data.

Following preprocessing, the dataset is **split into training and test subsets**, with 70% of the data allocated for model training and 30% reserved for validation. To mitigate the risk of overfitting, techniques such as stratified sampling and cross-validation are employed.

#### 2.3.3 Feature selection with genetic algorithms

Given the large number of variables (features) in the dataset and the relatively small number of patient cases, we used a feature selection process to reduce complexity and improve model performance. This was done using an advanced method called the **Non-dominated Sorting Genetic Algorithm II (NSGA-II)**, a type of evolutionary optimization that mimics natural selection to find the best combinations of features. NSGA-II helps us optimize two key goals at the same time:

• Minimize the number of selected features, while avoiding duplication or overlap in the information they provide.
• Maximize the performance of the classification model, aiming for high accuracy without overly increasing computational time.

The algorithm works by creating and evolving a “population” of feature sets across multiple generations. It includes several important steps:

• Non-dominated sorting: Groups different feature sets based on their performance, identifying the best ones across multiple objectives (accuracy and simplicity).
• Crowding distance: Maintains variety in the solutions, preventing the model from focusing too narrowly on one type of feature set.
• Tournament selection: Selects the most promising feature sets to be “parents” for the next generation based on performance and diversity.
• Genetic operations: Modifies and combines feature sets to create new ones. These include:
  ○ **Mutation rate**: Randomly alters a small number of features to introduce new possibilities.
  ○ **Elitism**: Keeps the best-performing feature sets unchanged for the next round to preserve progress.
  ○ **Annihilation**: Removes poorly performing sets to avoid wasting resources on unhelpful solutions.
  ○ **Elite replacement**: Replaces underperforming feature sets with stronger ones from earlier rounds.

#### 2.3.4 Classification models and hyperparameter tuning

To ensure robust performance, multiple **supervised classification** algorithms are tested, including:

• Decision Trees
• Random Forests
• Gradient Boosting Machines (GBM)
• k-Nearest Neighbors (KNN)
• Logistic Regression
• Support Vector Machines (SVM)

Hyperparameter tuning is conducted via **three-fold cross-validation**, employing either **Bayesian optimization** or **random search** depending on model complexity.

In addition to conventional tuning, the models are further optimized by modifying the objective score function to reflect the clinical relevance of classification errors. Specifically, four score functions—F1 and AUC for balanced datasets, and precision or recall for highly unbalanced datasets—are selectively applied. These functions are chosen based on the clinical importance of reducing either false positives or false negatives. This cost-sensitive approach ensures that the learning process aligns with the study’s clinical goals.

#### 2.3.5 Model integration and validation

The approach followed is structured into three specialized algorithms:

• First model for identifying ischemic stroke episodes.
• Second model for detecting hemorrhagic strokes.
• Third model for classifying large vessel occlusions (LVOs) among ischemic cases.

Once individual models are trained, their performance is evaluated on the test dataset by comparing predictions to the groundtruth. However, to improve global classification accuracy, the first two models (ischemic vs. hemorrhagic) are combined using a **Naive Bayes ensemble approach**. If an ischemic stroke is detected, the LVO classification model is subsequently applied to determine whether the patient requires thrombectomy. This multi-step approach enhances the reliability of prehospital stroke diagnosis and facilitates efficient triage for specialized treatment.

By following this comprehensive modeling strategy, the study ensures that predictive models are both accurate and generalizable, ultimately contributing to improved stroke care and patient outcomes.

#### 2.3.6 Ischemic and hemorrhagic classification model

The classification models for ischemic and hemorrhagic strokes were developed using the entire dataset, which presented a significant class imbalance. Given the higher prevalence of ischemic strokes compared to hemorrhagic strokes, the model required techniques to counteract bias and improve generalizability.

To address this imbalance, the **Synthetic Minority Oversampling Technique (SMOTE)** was applied. SMOTE generates synthetic samples of the minority class (hemorrhagic strokes) by interpolating existing data points, thereby increasing the representation of this class without merely duplicating observations. This process ensures a more balanced dataset, leading to improved model training and reducing the risk of bias toward the majority class.

For the development of two specialized models—one dedicated to ischemic stroke classification and the other to hemorrhagic stroke detection—both **precision and recall** were optimized to ensure a well-balanced performance. The focus on these metrics is crucial, as a model with high precision minimizes false positives, whereas high recall ensures that critical cases are not overlooked, particularly in a medical emergency setting where early stroke identification is vital.

The validation phase involved a rigorous comparison of **predicted labels vs. actual labels** to assess model performance. The evaluation metrics included:

• **Accuracy:** Measures the overall correctness of predictions.
• **Precision** (positive predictive value): Determines the proportion of correctly identified ischemic or hemorrhagic strokes out of all predicted cases.
• **Recall (sensitivity):** Evaluates the model’s ability to correctly identify all true ischemic and hemorrhagic cases.
• **F1-score:** Provides a harmonic mean between precision and recall, ensuring a balanced evaluation metric.

By employing SMOTE and optimizing key performance metrics, the developed models enhance the reliability of prehospital stroke classification, improving decision-making for emergency medical services (EMS) and facilitating timely intervention for stroke patients.

#### 2.3.7 LVO classification model

The large vessel occlusion (LVO) classification model was developed exclusively using ischemic stroke cases. Unlike the ischemic vs. hemorrhagic classification model, this dataset was already balanced, eliminating the need for oversampling or undersampling techniques. Instead, the focus was placed on optimizing feature selection and model performance to enhance predictive accuracy.

To assess model efficacy, the **Receiver Operating Characteristic (ROC) curve** was selected as the primary optimization metric. The ROC curve provides a comprehensive measure of the model’s diagnostic ability, evaluating its sensitivity and specificity across various classification thresholds. By maximizing the area under the ROC curve (AUC-ROC), the model ensures robust discrimination between LVO and non-LVO cases.

Validation of the LVO model was conducted through a multi-faceted approach. In addition to comparing predicted labels with actual clinical diagnoses, the model’s performance was benchmarked against the **Madrid Direct Scale**, a widely used prehospital assessment tool for determining thrombectomy eligibility. However, since the Madrid Direct Scale was originally designed to predict thrombectomy candidacy rather than LVO presence, its direct applicability to this study was limited.

To address this limitation, a **modified version of the Madrid Direct Scale** was developed specifically for LVO identification. The primary modification involved the exclusion of the **age factor**, which is typically included in the original scale to assess thrombectomy eligibility. Since age serves as a determinant for treatment eligibility rather than as a predictor of LVO itself, its removal allows for a more precise assessment of vascular occlusion status.

By refining the classification process and leveraging advanced performance metrics, this model enhances prehospital triage accuracy, ensuring that stroke patients with LVO receive timely intervention at thrombectomy-capable centers.

#### 2.3.8 Bayesian classification model

The Bayesian classification model is designed to enhance the accuracy of stroke classification by integrating probabilistic inference techniques. The LVO model is developed specifically using ischemic stroke episodes, as its primary focus is the identification of large vessel occlusions (LVOs). However, in contrast, the datasets for ischemic and hemorrhagic classification present a significant class imbalance due to the higher incidence of ischemic strokes.

To mitigate this imbalance, two highly specialized models are constructed:

• A model optimized for ischemic stroke prediction, trained to distinguish ischemic strokes from hemorrhagic episodes with a high degree of precision.
• A separate model optimized for hemorrhagic stroke detection, ensuring accurate identification of these less frequent but clinically significant cases.

These two models are then **combined using a Naive Bayes classification approach**. Naive Bayes is a probabilistic algorithm that assumes conditional independence between features, making it particularly suitable for integrating multiple models with distinct prediction targets. By applying this method, the system effectively uses the outputs of both models, leveraging their strengths to improve classification accuracy.

The probabilistic framework of Naive Bayes allows for dynamic weighting of model outputs based on prior probabilities, reducing the influence of misclassified instances and enhancing overall robustness. This approach ensures that the classification system remains adaptable, providing reliable prehospital stroke detection and supporting emergency decision-making processes.

By integrating Bayesian inference into the classification pipeline, this model enhances stroke subtype differentiation, ultimately improving prehospital triage and optimizing patient management strategies.

Bayesian model is trained with a sample defined by the features needed for the previous models (ischemic, and hemorrhagic), yielding a larger sample, that will be splitted to obtain two new samples training and test sample to avoid overfitting.

## 3 Results

### 3.1 Data analysis

In the master dataset, sex and age are the only demographic variables available; all other features are clinical in nature. As a preliminary step before model development, these demographic variables are analyzed to assess their distributions and their potential associations with the primary outcome labels: stroke type (ischemic vs. hemorrhagic) and large vessel occlusion (LVO) status (yes vs. no).

**Figure 4:**
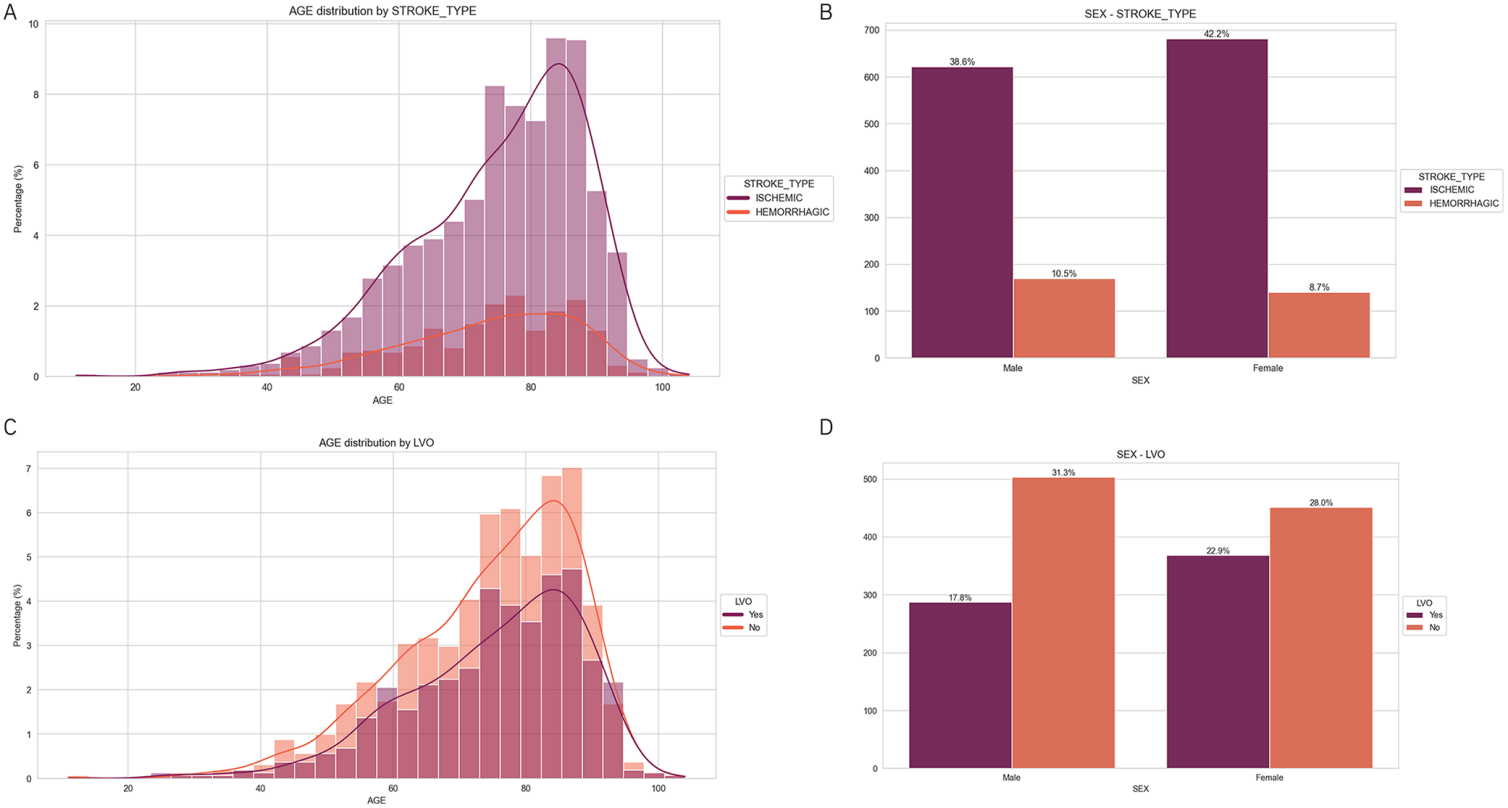
Demographic distributions by stroke type and large vessel occlusion (LVO). **(A)** Age distribution stratified by stroke type, showing higher prevalence of ischemic strokes in older individuals. **(B)** Sex distribution by stroke type, with a higher proportion of ischemic strokes in both males and females. **(C)** Age distribution by LVO status, indicating a slightly younger peak in non-LVO cases. **(D)** Sex distribution by LVO status, with a higher proportion of non-LVO cases in both sexes.

The top-left panel of Figure 4 shows that ischemic strokes are more prevalent in older patients, with a peak incidence between ages 75–85. Hemorrhagic strokes occur less frequently and show a slightly flatter distribution across age groups. To statistically assess this difference in age distributions between stroke types, Shapiro–Wilk test is used. Results indicated a violation of the normality assumption in both stroke-type groups (all p<0.05), warranting the use of a non-parametric test. Therefore Mann–Whitney U test (Wilcoxon rank-sum test) is applied, which yielded a U statistic of 216,653.5 and a two-tailed p-value of 0.051. At the conventional 5% significance level, the difference in age distributions between ischemic and hemorrhagic stroke patients did not reach statistical significance. However, at the 10% significance level, this difference is considered statistically significant, suggesting a borderline effect of age on stroke type.

The top-right panel of Figure 4 displays the sex distribution by stroke type. Ischemic strokes are more common than hemorrhagic strokes in both males and females. To further examine the association between patient sex and stroke type, a $\chi^{2}$ test of independence was implemented. The test produced a $\chi^{2}$ statistic of 4.7748 and a p-value of 0.0289. As p<0.05, we reject the null hypothesis of independence and conclude that there is a statistically significant, although small, association between sex and type of stroke.

The bottom left panel of Figure 4 shows the age distribution stratified by LVO status. Both LVO-positive and LVO-negative cases exhibit a right-skewed age distribution, with slightly higher frequencies of LVO-negative cases at older ages. Again, due to non-normality (confirmed via Shapiro–Wilk test, p<0.05), Mann–Whitney U test is used to compare the groups. Results indicated non-normal distributions (all p<0.05). Therefore, a Mann–Whitney U test was used to compare age distributions between LVO-positive and LVO-negative patients. The resulting U statistic was 325,088.5, with a two-tailed p-value of 0.1906. This indicates no statistically significant difference in age distributions between patients with and without LVO at the 5% significance level.

Finally, the bottom right panel of Figure 4 illustrates the distribution of the LVO status by sex. LVO-negative cases are more common in both sexes, but notably, females have a higher proportion of LVO-positive cases compared to males. The relationship between sex and LVO was assessed using a $\chi^{2}$ test of independence. The $\chi^{2}$ test resulted in a test statistic of 11.9786 and a p-value of 0.000538. Given that this p-value is well below 0.05, we reject the null hypothesis and conclude that there is a statistically significant association between sex and LVO status.

### 3.2 Ischemic and hemorrhagic models development

In order to develop the most effective predictive models for ischemic and hemorrhagic stroke classification, the methodological pipeline described in Section 2.3 was implemented. This process involved generating multiple datasets with varying characteristics to evaluate model performance under different conditions. Specifically, three distinct datasets were created, each differing in the number of variables and observations:

• A dataset consisting of 94 variables and 92 observations.
• A dataset with 46 variables and 632 observations.
• A dataset containing 27 variables and 1,341 observations.

For each of these datasets, a Genetic Algorithm (GA) was applied to optimize both the selection of features and the performance of the predictive models. The GA aimed to identify the most relevant subset of features while maximizing the selected performance metrics.

For the ischemic stroke classification model, the best results were achieved using a Decision Tree classifier optimized for recall, prioritizing the correct identification of actual positive cases. The hyperparameters that produced the best performance for the Decision Tree were:

• **Criterion:** Gini index
• **Maximum depth:** 2
• **Minimum samples per leaf:** 10

n contrast, the most effective model for hemorrhagic stroke classification is a K-Nearest Neighbors (KNN) classifier, optimized for precision—emphasizing the accuracy of positive predictions by minimizing false positives. The model achieved its best performance with the following configuration:

• **Number of neighbors (k):** 2

The predictive performance of the ischemic and hemorrhagic models was assessed based on several key metrics, including training best metric performance, area under the curve (AUC), test accuracy, and test F1-score. The results are summarized in Table 1, where it can be observed the comparison between the metrics during training and with a external sample testing. The ischemic stroke classification model, optimized for recall, achieved a recall of 86.11%, with a cross-validation mean of 0.861, a standard deviation of 0.0393, and a 95% confidence interval of [0.764, 0.959]. The hemorrhagic stroke classification model, optimized for precision, achieved a precision of 79.63%, with a cross-validation mean of 0.796, a standard deviation of 0.0729, and a 95% confidence interval of [0.615, 0.977].

**Table 1:** Comprehensive performance metrics of the ischemic and hemorrhagic stroke models across both training and testing phases.

Metric	Ischemic model	Hemorrhagic model
Training metrics		
Train best metric	86.11%	79.63%
Train area under the curve (AUC)	91.29%	98.92%
Testing metrics		
Test area under the curve (AUC)	66.67%	89.58%
(95% CI)	(44.94%–61.19%)	(45.18%–68.27%)
Test F1-score	91.67%	73.68%
(95% CI)	(80.05%–89.40%)	(77.17%–86.82%)
Test accuracy	85.71%	64.29%
(95% CI)	(63.25%–87.69%)	(64.74%–78.20%)
Negative predictive value (negative precision)	50.00%	28.57%
(95% CI)	(34.65%–46.90%)	(13.63%–41.93%)
Positive predictive value (positive precision)	91.67%	100.00%
(95% CI)	(80.22%–89.26%)	(79.64%–91.33%)
Specificity (negative recall)	50.00%	100.00%
(95% CI)	(45.00%–72.05%)	(79.64%–91.33%)
Sensitivity (positive recall)	91.67%	58.33%
(95% CI)	(72.13%–88.76%)	(20.00%–56.00%)

The table presents model performance during training (top section) and validation on the testing sample (bottom section). Reported metrics include accuracy, AUC, sensitivity, specificity, predictive values, and class-wise F1-scores. Where available, 95% confidence intervals (CI) are included.

Among the three datasets analyzed, the highest performance for both ischemic and hemorrhagic models was achieved when using the dataset containing 94 variables and 92 observations. After applying feature selection using GA, the ischemic model was optimized using only 18 variables, which accounted for 18.56% of the original dataset. The hemorrhagic model, on the other hand, required just 7 variables, representing a mere 7.22% of the total features initially available.

**Figure 5:**
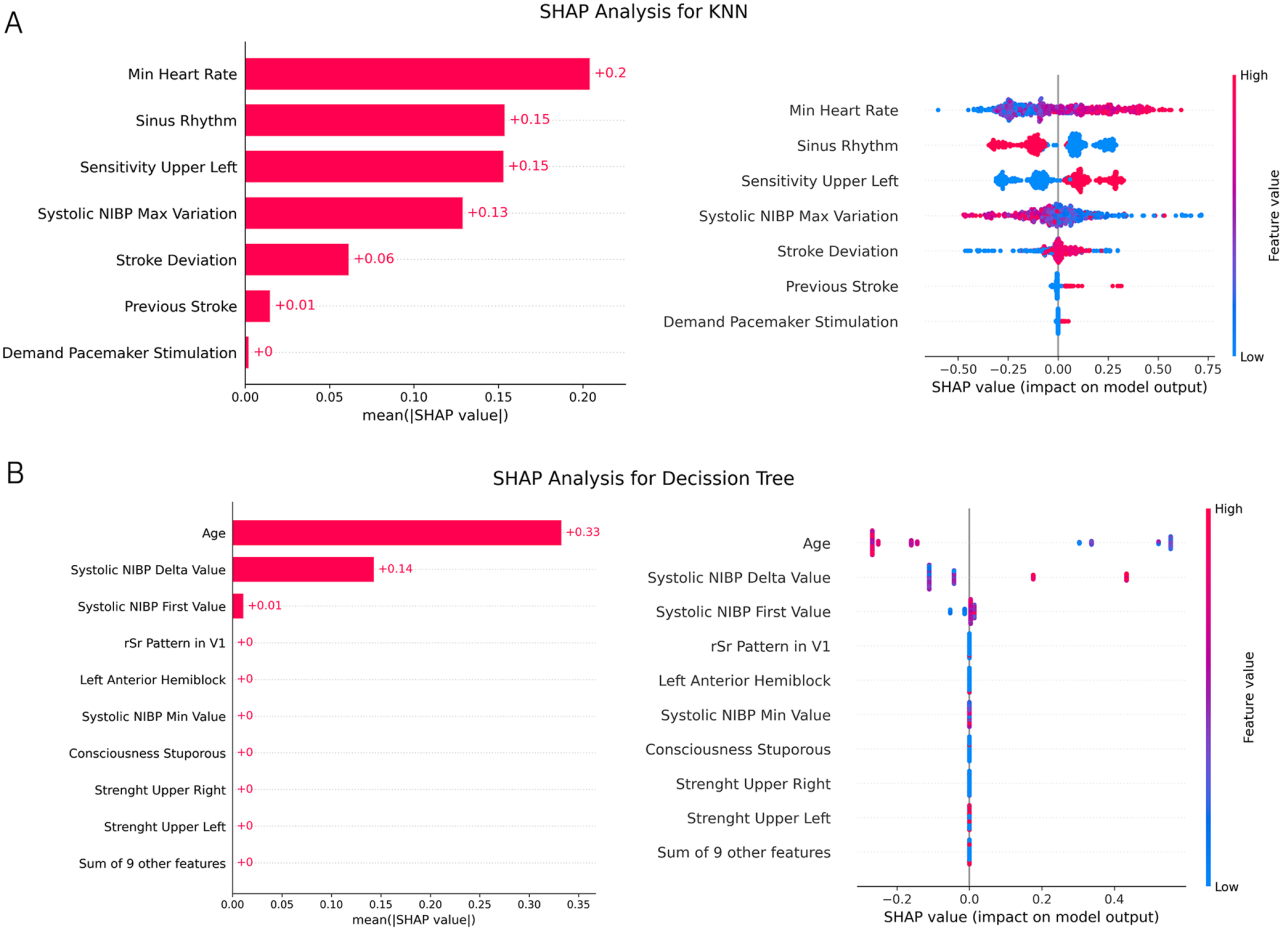
**SHAP analysis** for ischemic and hemorrhagic stroke prediction models. **(A)** SHAP analysis for the K-Nearest Neighbors (KNN) model applied to hemorrhagic stroke prediction. The left panel shows the global feature importance ranked by the mean absolute SHAP values. The right panel presents a SHAP summary plot, illustrating the distribution of SHAP values for each feature across all observations. Color indicates feature value (red = high, blue = low), and position on the x-axis indicates impact on model output. **(B)** SHAP analysis for the Decision Tree model used in ischemic stroke prediction. The left panel displays global feature importance based on the mean absolute SHAP values, with *Age* and *Systolic NIBP Delta Value* showing the highest contributions. The right panel shows the corresponding SHAP summary plot, visualizing the feature influence distribution across individual predictions.

The most representative features for each model are presented in Figure 5 calculated by the mean value of SHAP for each observation. The hemorrhagic model (KNN) show that almost all 7 variables have a representative impact. However, when analyzing the decission tree results it can be observed a diminished relevance of certain features, as quantified by their mean SHAP values, This can be attributed to the model’s inherent hierarchical partitioning strategy. Dominant predictors, such as “Age” and “Systolic NIBP Delta Value,” effectively reduce data impurity at higher decision nodes. This initial, potent stratification significantly constrains the remaining variance within subsequent sub-nodes, thereby limiting the scope and impact of other features for further splits. Consequently, while potentially contributing to overall model performance within specific data partitions, the average individual contribution of these less impactful features across the entire dataset is attenuated, resulting in lower mean SHAP values. In same figure also illustrates a SHAP analysis conducted on an independent validation sample, which is larger than the sample used for model training and testing. This independent dataset was constructed by selecting the minimal number of variables necessary, thereby increasing the number of available observations.

To assess model robustness and generalization, we evaluated performance on an independent test set using precision, recall, and F1-score. To quantify variability, we applied a bootstrap approach with 1,000 resamples to estimate confidence intervals (CIs) for these metrics. Results, detailed in Table 1, show that the ischemic model has strong positive predictive power, while the hemorrhagic model achieves perfect precision but lower sensitivity.

The results indicate that the Decision Tree model effectively classifies ischemic strokes, while the KNN model is more suitable for hemorrhagic stroke classification. The relatively high specificity and sensitivity observed in the ischemic model suggest a strong predictive ability, whereas the hemorrhagic model, despite achieving perfect positive predictive value, exhibited lower sensitivity.

**Figure 6:**
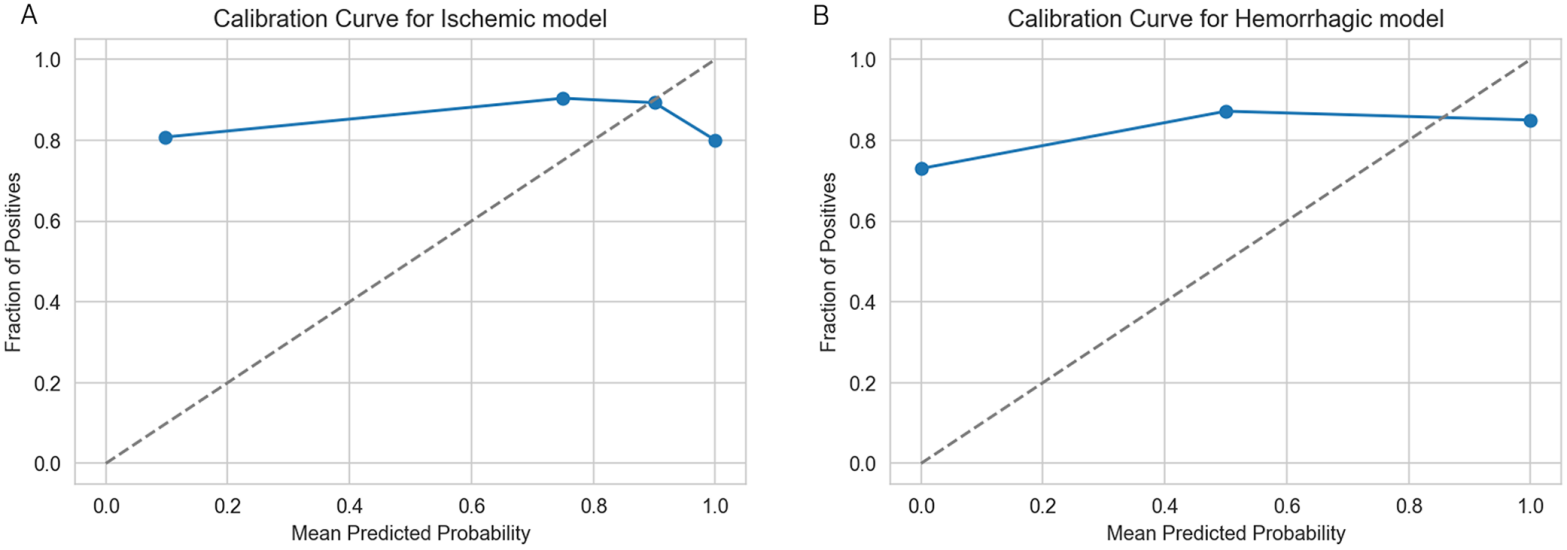
Calibration curves for stroke type models. **(A)** Calibration curve for ischemic model. **(B)** Calibration curve for hemorrhagic model. The x-axis represents the mean predicted probability, and the y-axis represents the observed fraction of positives. The diagonal dashed line indicates perfect calibration.

Calibration curves were calculated to evaluate how well the predicted probabilities from the ischemic and hemorrhagic stroke models align with actual outcomes. Unlike metrics such as accuracy or AUC, calibration assesses the reliability of probability estimates, which is essential for clinical decision-making. Well-calibrated models enable more trustworthy risk communication and treatment planning. Results obtained are in Figure 6 showing that both models demonstrate reasonable calibration across probability bins, with slight overestimation observed at higher predicted probabilities.

Beyond conventional performance metrics, calibration assessments, and validation results, evaluating potential biases in machine learning models is critical—particularly in clinical contexts where predictions may influence medical decisions or patient outcomes. In this analysis of the stroke subtype classification models, the presence of bias with respect to input variables was assessed. As shown in Figure 5, the hemorrhagic stroke model only includes clinical features directly related to symptoms or diagnostic indicators. Therefore, by design, it cannot express bias toward any demographic group, as no sensitive or demographic variables are part of the model’s feature set.

In contrast, the ischemic stroke model incorporates age as one of its predictive variables. Age is inherently linked to stroke prevalence and may introduce bias into the model’s behavior. This relationship was preliminarily examined in Section 3.1, where statistical analysis suggested a borderline significant difference in age distributions between ischemic and hemorrhagic stroke populations, dependent on the threshold chosen for significance. To further investigate this, age was discretize into 10 quantile-based bins and applied a chi-squared test to test for independence between age group and stroke type. The results ($\chi^{2}=23.27$, p=0.0056, df=9) showed that the null hypothesis (p<0.01) can be rejected, confirming that stroke type is significantly associated with age bin in our dataset. To evaluate model fairness across age groups, three key metrics are studied:

• Selection Rate: the proportion of individuals in a group who are predicted as positive (i.e., classified as ischemic stroke).
• True Positive Rate (TPR): the proportion of actual positives correctly identified—measuring the model’s sensitivity within each group.
• False Positive Rate (FPR): the proportion of actual negatives incorrectly identified as positives—indicating the model’s error rate per group.

These metrics are calculated across the 10 age bins, as shown in Table 2. The Demographic Parity Difference, which measures the disparity in selection rates across groups, is approximately 0.885. This large gap is driven by nearly universal positive predictions in bins 1–3 (100% selection rate) vs. much lower rates in older bins. The Equalized Odds Difference, which evaluates differences in both TPR and FPR across groups, is 1.0, reflecting extreme variations between the youngest and oldest age bins (FPR = 100% for bin 1 and FPR = 0% for bin 10).

**Table 2:** Fairness analysis across age bins for the ischemic stroke model based on selection rate, sensitivity (true positive rate), and false positive rate.

Age bin	Selection rate	TPR	FPR
1	100.0%	100.0%	100.0%
2	100.0%	100.0%	100.0%
3	100.0%	100.0%	100.0%
4	81.5%	74.1%	86.8%
5	13.1%	29.4%	6.8%
6	11.5%	10.3%	13.0%
7	33.3%	48.4%	20.0%
8	17.7%	16.7%	19.2%
9	14.8%	18.8%	10.3%
10	16.0%	26.7%	0.0%

These results show that the model learned to consistently predict positive for younger individuals (bins 1–3 and partially bin 4), likely because those bins contained a higher proportion of stroke cases—particularly hemorrhagic strokes—in the training data. Conversely, bins 5–6 (middle-aged individuals) saw few positive predictions and almost no false positives, suggesting lower prevalence in this group and a more conservative model behavior. In the older bins (7–10), the model’s behavior becomes more stable, with selection rates and error rates falling within a moderate and consistent range. This model bias reflects the underlying population imbalance rather than intrinsic algorithmic unfairness. The model’s decisions are consistent with the observed distribution of stroke cases in the dataset. To reduce this bias, a possible mitigation strategy is to apply reweighting techniques to reduce overrepresentation of younger individuals and increase the influence of underrepresented groups, such as older adults. This may affect overall accuracy, as the model currently reflects the prevalence patterns in the training data.

### 3.3 LVO model development

To construct the optimal model for Large Vessel Occlusion (LVO) classification, the methodological pipeline outlined in Section 2.3 was employed. Initially, four distinct datasets were generated depending on the number of the percentage of informed variables, each varying in the number of observations:

• 129 variables and 21 observations
• 94 variables and 94 observations
• 46 variables and 437 observations
• 27 variables and 1,350 observations

For each dataset, a Genetic Algorithm (GA) was utilized to optimize both the feature selection process and the classification model. The most effective model identified was a Gradient Boosting classifier optimized for AUC with the following hyperparameters:

• **Number of estimators:** 600
• **Maximum depth:** 4
• **Learning rate:** 0.1

The performance metrics of the selected model are summarized in image Table 3, highlighting its training results. The Large Vessel Occlusion (LVO) classification model, optimized for AUC, achieved an AUC of 59.84%, with a cross-validation mean of 0.598, a standard deviation of 0.1411, and a 95% confidence interval of [0.448, 0.949].

**Table 3:** Comprehensive performance metrics of the LVO model, Madrid Direct standard approach, and its modified version, across both training and testing phases.

Metric	LVO model	Madrid direct	Madrid direct modified
Training metrics			
Train best metric	59.84%	–	–
Train area under the curve (AUC)	100.00%	–	–
Testing metrics			
Test area under the curve (AUC)	74.31%	–	–
(95% CI)	(70.83%–88.67%)		
Test accuracy	70.83%	–	–
(95% CI)	(66.25%–84.61%)		
Test F1-score (positive class)	75.86%	54.55%	58.33%
(95% CI)	(66.67%–84.61%)		
Negative predictive value (negative precision)	85.71%	57.14%	58.33%
(95% CI)	(68.57%–90.63%)		
Positive predictive value (Positive precision)	64.71%	60.00%	58.33%
(95% CI)	(59.52%–82.60%)		
Specificity (negative recall)	50.00%	60.67%	58.33%
(95% CI)	(55.52%–80.00%)		
Sensitivity (positive recall)	91.67%	50.00%	58.33%
(95% CI)	(71.10%–92.10%)		

The table presents training metrics (top section) for the LVO model and testing metrics (bottom section) for all three approaches. Metrics include AUC, accuracy, predictive values, sensitivity, specificity, and class-wise F1-scores, with 95% confidence intervals (CI) shown when available.

**Figure 7:**
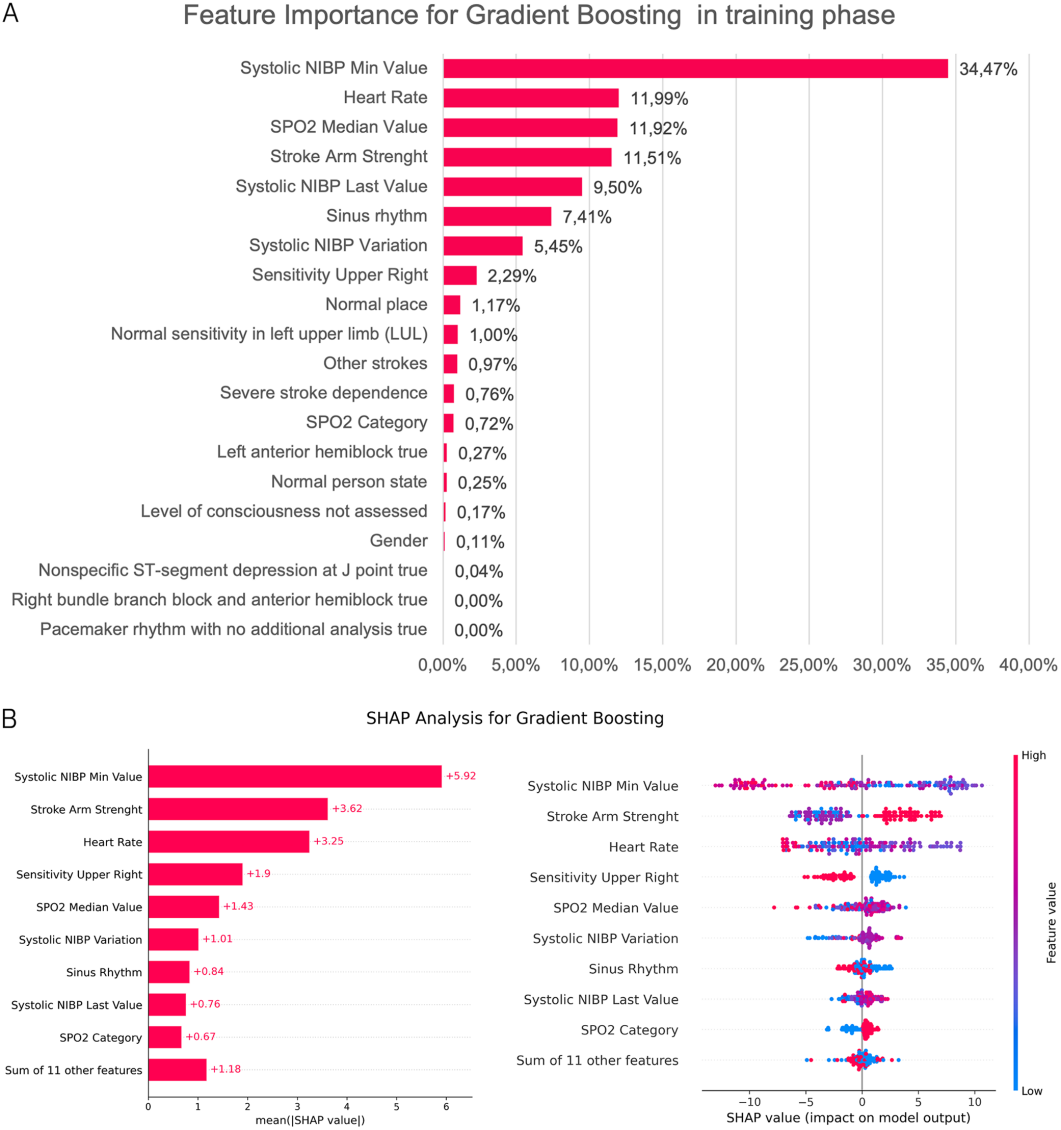
Feature importance and SHAP analysis for LVO model. **(A)** Feature importance derived from the Gradient Boosting model during the training phase, quantified by the relative contribution of each feature to the model’s decision-making process. **(B)** SHAP analysis of the same model. The left panel shows global feature importance ranked by the mean absolute SHAP values across all observations. The right panel presents the SHAP summary plot, illustrating the distribution and direction of each feature’s impact on individual predictions. Color represents feature values (red = high, blue = low), while horizontal position indicates the effect on model output.

Among the four datasets evaluated, the dataset containing 94 variables yielded the highest model performance. Following feature selection, 20 variables were retained, representing 20.83% of the original feature set. The final set of selected features, along with their respective importance scores, is presented in Figure 7. The same figure also illustrates a SHAP analysis conducted on an independent validation sample, which is larger than the sample used for model training and testing. This independent dataset was constructed by selecting the minimal number of variables necessary, thereby increasing the number of available observations. A comparison of both analyses revealed that the most influential variables included minimum systolic blood pressure, heart rate, arm strength, and the median SpO2. To further clarify feature contributions, SHAP values were computed for each individual observation, providing insights into the distribution and directional impact of each feature on the model’s predictions.

The model’s predictive capability was further validated against an independent test sample. To quantify variability, a bootstrap approach was implemented with 1,000 resamples to estimate confidence intervals (CIs) for these metrics. Table 3 compares the LVO model’s performance against the Madrid Direct and Madrid Direct Modified approaches, emphasizing key evaluation metrics such as precision, recall, and F1-score. These results indicate that the Gradient Boosting model provides a strong predictive advantage over the comparative methods, particularly in terms of sensitivity and negative predictive value.

**Figure 8:**
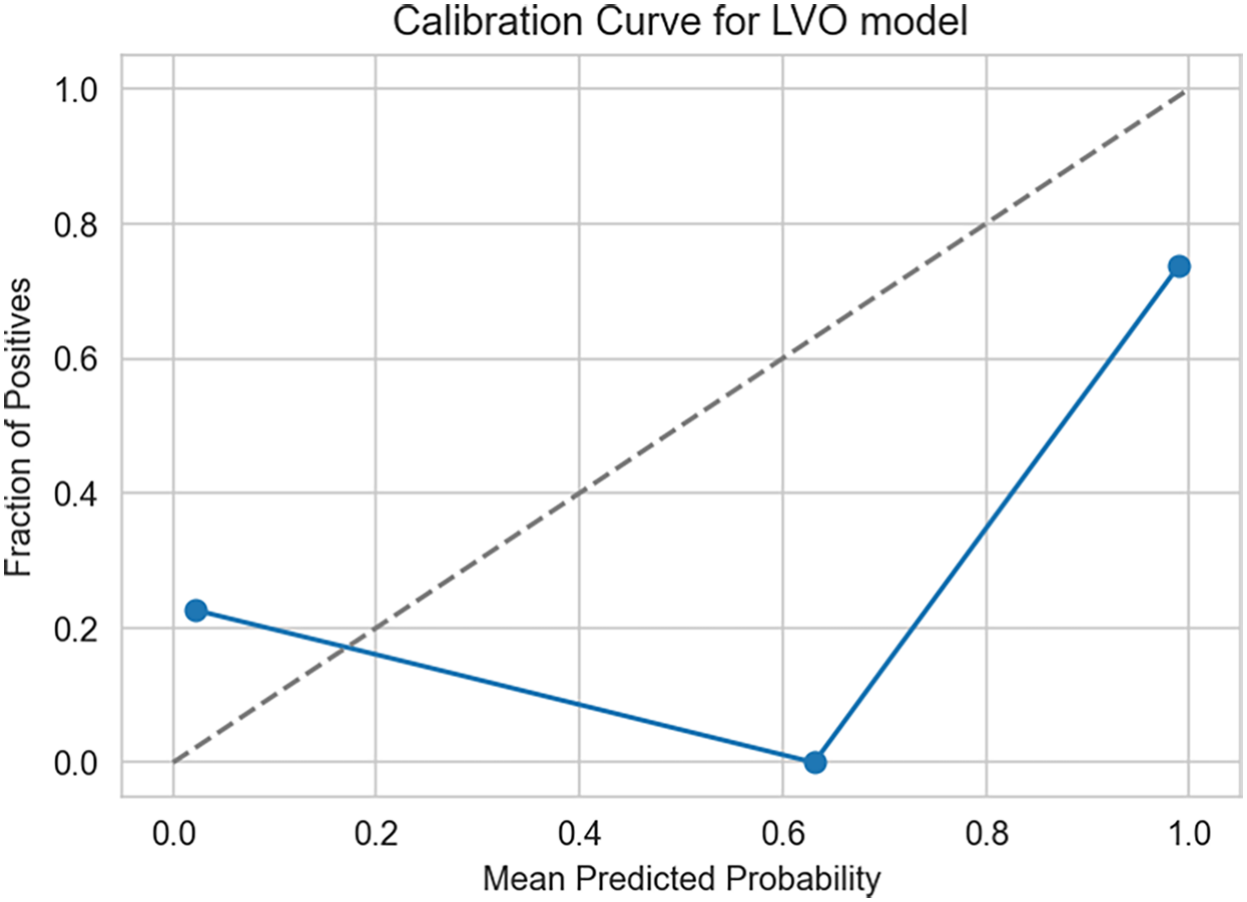
Calibration curve for LVO model. The x-axis represents the mean predicted probability, and the y-axis represents the observed fraction of positives. The diagonal dashed line indicates perfect calibration.

Furthermore, the calibration curve is also calculated for the LVO model to better understand how the predicted probabilities align with actual outcomes. Results obtained are in Figure 8. The model shows good reliability in high-confidence predictions, where it tends to be accurate.

Finally, in addition to standard performance metrics, validation results, and calibration curves, it is essential to assess the potential biases present in the model. For the LVO detection model, sex is the only demographic variable among the input features that could introduce bias, as all other variables are related to clinical symptoms or findings (variables in Figure 7). Accordingly, the impact of sex on model performance was specifically evaluated.

The model shows a Demographic Parity Difference of 11.25% and an Equalized Odds Difference of 8.13%, when stratified by sex. Females are predicted to have a positive LVO outcome 59.2% of the time, compared to 47.9% for males, indicating an 11.3 percentage point gap. However, this difference mirrors the underlying data distribution as the actual prevalence of LVO in females is 57%, vs. 43% in males, which is a difference that is statistically significant as reported in Section 3.1.

With respect to classification performance, the True Positive Rate (TPR) is 83.1% for females and 75.0% for males, yielding an 8.1 percentage point difference, while the False Positive Rate (FPR) is 37.4% for females and 34.7% for males, a 2.7 percentage point difference. These differences are within acceptable thresholds and indicate that the model maintains relatively equitable performance across sexes. Therefore, while disparities in prediction rates exist, they reflect the real-world distribution of LVO cases in the population rather than any intrinsic bias introduced by the model. As a conclusion, the model is not biased with respect to sex, and the observed differences are attributable to genuine demographic patterns in the dataset.

### 3.4 Final model

The final step in our modeling process involves implementing the Naïve Bayes algorithm, which is particularly effective in handling classification problems with imbalanced datasets. Since class imbalance can lead to biased predictions that favor the majority class, optimizing the prior probability is crucial to ensuring that the model fairly represents both ischemic and hemorrhagic cases. Additionally, proper optimization prevents overfitting to the training data and enhances the overall predictive performance. The final sample used to train the Bayesian model consists of patients for whom the minimal set of required variables to predict the probability of ischemic and hemorrhagic stroke is available. This criterion allows us to include a larger dataset, totaling 489 patients. From this sample, 70% (342 patients) is used for model training, while the remaining 30% (147 patients) is reserved exclusively for validation to assess model performance on unseen data.

**Figure 9:**
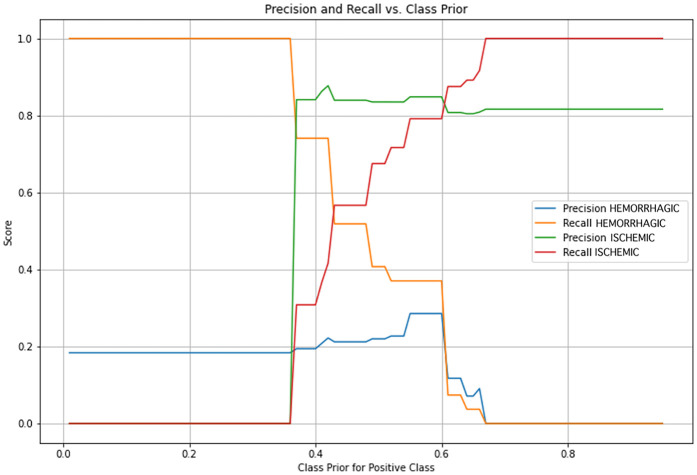
Optimization of class prior for Bayesian classification model. Precision and recall curves for both hemorrhagic and ischemic stroke classes are plotted against varying values of the class prior assigned to the positive class in a Bayesian model. The objective is to identify an optimal prior probability that balances precision and recall for each class.

To achieve the best possible classification accuracy, a grid search approach was applied to identify the optimal prior parameter. This technique systematically evaluates different prior probability values and selects the one that yields the highest predictive performance for the positive class. Figure 9 illustrates the optimization process for the prior parameter in the Naïve Bayes algorithm.

Through this optimization process, a prior of 0.6 was determined to be the most effective for the positive class, meaning that the model assigns a slightly higher probability to ischemic cases. This adjustment improves sensitivity towards ischemic predictions without excessively compromising specificity. The performance metrics obtained from the optimized Naïve Bayes model for the testing sample are summarized in Table 4.

**Table 4:** Bayesian model performance metrics—precision and recall—for positive class (ischemic) and negative class (hemorrhagic).

Metric	Bayesian model
Negative predictive value (negative precision)	28.57%
Positive predictive value (positive precision)	84.84%
Specificity (negative recall)	37.03%
Sensitivity (positive recall)	79.16%

Metrics calculated in testing sample.

Results indicate a strong predictive ability for ischemic cases, with an positive precision of 84.84% and a recall of 79.16%. However, the hemorrhagic classification remains a challenge, with a relatively lower precision and recall. This is expected given the class imbalance in the dataset, where ischemic strokes are more prevalent than hemorrhagic ones.

To comprehensively assess the model’s effectiveness, an evaluation was conducted using three different patient samples. These samples were carefully selected to analyze the generalizability of the model across various clinical scenarios:

• **Ischemic by bayes:** Patients classified as ischemic using the Naïve Bayes model based on their input features.
• **Real ischemic:** Patients with a confirmed ischemic stroke diagnosis from medical records.
• **Complete sample:** A dataset containing both ischemic and hemorrhagic patients to simulate a real-world clinical setting.

To ensure the reliability of the analysis, only patients with complete and valid data for the LVO model were included. This criterion was essential to maintain consistency in the evaluation process and to avoid biases due to missing information.

To further examine the model’s predictive capabilities, we evaluated the performance of the LVO model across these three patient groups. The LVO model was applied separately to the ischemic predictions made by the Naïve Bayes model, to real ischemic cases, and to the complete dataset. This analysis allowed us to compare how the LVO model behaves under different input conditions and determine whether using the Naïve Bayes model as a pre-filter impacts overall performance.

To further contextualize these results, the LVO model’s performance was compared with a reference model. The comparison highlights potential areas of improvement and helps determine whether the Naïve Bayes-based filtering enhances diagnostic accuracy. In Table 5, the analysis is conducted. The first section defines the patient groups, with 198 patients included in the first analysis, 266 in the second, and 320 in the final analysis. The second section details the results of the LVO model. The final section presents the results obtained by the referenced model (Madrid Direct Modified). In this section, a color-coded scheme is used to compare the performance of the reference model with the LVO model: green indicates that the reference model achieved significantly better results than the LVO model (>5%), orange represents comparable performance (−5% to 5%), and red signifies that the reference model performed significantly worse than the LVO model (<−5%).

**Table 5:** Comparison of relevant metrics in three different populations.

	Ischemic by bayes		Real ischemic		Complete sample	
Sample selected to conduct results comparison						
Patients	323		523		523	
Required variables	198		320		320	
True ischemic	N/A		266		N/A	
LVO model results						
Metrics	No	Yes	No	Yes	No	Yes
Precision	77.60%	59.00%	79.60%	74.00%	82.00%	60.60%
Recall	63.40%	74.00%	72.50%	80.95%	64.00%	80.19%
F1-score	69.00%	66.30%	75.92%	77.37%	72.02%	69.08%
Madrid direct modified						
Metrics	No	Yes	No	Yes	No	Yes
Precision	73.10%	60.00%	70.00%	70.63%	75.00%	60.00%
Recall	68.00%	65.00%	72.59%	67.94%	69.00%	67.00%
F1-score	70.80%	62.00%	71.27%	69.26%	72.10%	64.00%

Ischemic episodes identified by Bayesian model, Real ischemic episode, and complete sample including ischemic and hemorrhagic episodes. This table gathers the final results in an independant sample and compares results to the Madrid Direct Modifies (Red—Significant decrease in accuracy, Orange—Slight change in accuracy).

## 4 Discussion

### 4.1 Principal findings

The primary objective of this study is to develop a robust framework for detecting Large Vessel Occlusions (LVO) during Emergency Medical Services (EMS) interventions to improve the identification of thrombectomy candidates and avoid second transfers that increase the time until the patient is treated. To achieve this goal, three distinct models were designed, each leveraging a subset of the most critical features from a comprehensive set of demographic, clinical, and hemodynamic characteristics. Table 6 provides a summary of all developed models to facilitate a better understanding. Furthermore, the utilization of evolutionary algorithms enabled the simultaneous optimization of both model precision and the number of variables employed.

**Table 6:** Model card summary including intended use, best model and parameters, metric use to be optimized, test metrics, limitation and the number of variables finally included.

Attribute	Ischemic model	Hemorrhagic model	LVO model
Intended use	Classification of stroke subtype focusing on ischemic identification	Classification of stroke subtype focusing on hemorrhagic identification	Classification of large vessel occlusion (LVO) in ischemic episodes
Best model and parameters	Decision tree Gini criterion max_depth=2 min_samples=10	K-Nearest Neighbors k=2	Gradient Boosting n_estimators=600 max_depth=4 learning_rate=0.1
Optimization metric	Recall	Precision	AUC
Test performance	AUC: 66.67% F1: 91.67%	AUC: 89.58% F1: 73.68%	AUC: 74.31% F1: 75.86%
Limitations	Biased towards younger people	Low NPV and sensitivity	Needs improved calibration in middle probability ranges
Number of variables	18	7	20

The introduction of the Non-dominated Sorting Genetic Algorithm II (NSGA-II) optimization technique played a pivotal role in enhancing the performance of the Machine Learning (ML) model. Leveraging the search capabilities of Genetic Algorithms (GA), the model’s input features were fine-tuned, resulting in improved classification accuracy. The GA’s exploration of a vast solution space allowed for the identification of the optimal feature set among the initial 271 variables. The implementation of NSGA-II not only optimized the number of variables used but also ensured the best possible model performance. This methodology facilitated the creation of a comprehensive set of experiments, wherein input variables, model selection, hyperparameters, and NSGA-II parameters were optimized.

The ischemic model, which utilized a decision tree, achieved a precision of 91.67% and a recall of 91.67%. However, the specificity obtained was relatively low, at 50%. These results indicate that the model exhibits a high capacity for identifying ischemic strokes, with a high rate of false positives. This suggests that while the model is effective in detecting true positives, it may also generate a significant number of false positive.

In contrast, the hemorrhagic model demonstrated the lowest quality in terms of precision results. This can be attributed to the inherent imbalance present in the data, where only 10%–15% of the sample consisted of hemorrhagic cases. The model achieved a recall of 100% with a precision of 28.57%, indicating that it is capable of identifying all hemorrhagic episodes, at the expense of including a high number of false positives. This highlights the challenges associated with developing models for rare events, where the risk of false positives can be substantial.

These two models are the foundation to apply the final LVO model. This model has shown promising results. The model obtained a recall of 91.67% and a precision of 64.71%. To contextualize these results, the Madrid Direct modified scale was used as a baseline, which is a modification of the original scale to predict LVO instead of thrombectomy. A comparison of the model results revealed an increase in all performance metrics, except for specificity, which was reduced by 8% as shown in Table 3. However, sensitivity was improved by more than 30%. From a clinical perspective, this enhancement means better identification of actual LVO cases, which can reduce unnecessary inter-hospital transfers and potentially shorten the time to treatment. On the other hand, the slight decrease in specificity may lead to an increased number of false positives, potentially resulting in more patients being referred for further evaluation, some of whom may not ultimately require specialized care.

In particular, the LVO model utilizes only 20 out of the 271 original variables. The most representative variables obtained include blood pressure, heart rate, oxygen saturation, and arm movement. Clinically, the inclusion of some of these variables aligns with those used in widely adopted neurological scales such as the NIHSS (17), while also incorporating novel hemodynamic variables that provide additional insights. These variables facilitate stroke diagnosis, as most of them are automatically obtained from monitors, rather than relying on traditional clinical variables that can be subjective, such as arm movement. The selection of these variables highlights the importance of leveraging objective, quantifiable measures in the development of predictive models for LVO.

The implementation of the models in isolation do not have the expected value, being need the development of a pipeline to validate all models. To achieve this, the ischemic and hemorrhagic models were integrated with a Bayesian model to obtain the final decision. The pipeline first determines whether a case is ischemic or not, and subsequently, the LVO model is applied if an ischemic episode is identified. This approach enables a more comprehensive evaluation of the models’ performance.

To assess the final performance of the LVO model, three distinct samples were defined: ischemic cases identified by the Bayesian pipeline, actual ischemic cases, and a complete sample comprising both ischemic and hemorrhagic cases. Results of this evaluation, shown in Table 5 revealed that the complete pipeline accumulated errors from multiple models, including the hemorrhagic model, resulting in worse performance compared to the LVO model applied to the entire sample. Specifically, the complete pipeline achieved a precision of 59% and a recall of 74%, whereas the LVO model applied to the whole sample yielded a precision of 60.60% and a recall of 80.19%.

However, the results obtained from the real ischemic sample were slightly better than the results obtained when applied to the whole sample. Positive precision is 74% vs. 60.60%, and negative recall, is 72.50% vs. 64%. The positive recall obtained was similar, with 80.95% vs. 80.19%. These findings suggest that the inclusion of hemorrhagic episodes in the analysis led to a decrease in performance, but the results were still superior to those obtained by implementing the complete pipeline. This indicates that the Bayesian pipeline, while useful for integrating the models, may introduce additional errors that negatively impact the overall performance of the LVO model. The main limitation of this pipeline lies in the weakness of the hemorrhagic model, primarily due to the limited number of episodes in the current sample. This issue could be addressed by increasing both the overall dataset size and the number of hemorrhagic cases.

The efficacy of the LVO model is underscored by a comparative analysis with the Madrid Direct Modified, which serves as the baseline paradigm in this study. An outstanding observation is that the implementation of the LVO model yields an enhancement in performance metrics across all three samples. Notably, a granular examination of the entire pipeline reveals a significant improvement in positive recall, with an increase of approximately 10% from a baseline value of 65% obtained via the prehospital scale to a value of 74% achieved by the LVO model. This improvement is further accentuated when the LVO model is applied to the entirety of the sample, resulting in a 13% increase in positive recall, from a baseline value of 67% obtained by the scale to a value of 80% obtained by the model. In addition, an increase in negative precision was observed, with values going from 75% to 82%.

These findings collectively suggest that the integration of Machine Learning (ML) techniques can precipitate a notable improvement in the diagnostic accuracy of stroke in the context of Emergency Medical Services (EMS), with the incorporation of objective variables serving as a pivotal factor in this enhancement. The utilization of ML models, such as the LVO model, can potentially augment the precision of stroke diagnosis, thereby facilitating more efficacious and timely interventions. Ultimately, the results of this study underscore the potential benefits of leveraging ML techniques in the realm of Emergency Medicine, with a particular emphasis on the diagnosis and management of stroke.

However, to better understand the clinical relevance of this study, it is essential to assess its impact from the patient’s perspective. Under the current Madrid Stroke Code, Stroke Units are geographically designated hospitals equipped to perform thrombectomies and manage large vessel occlusion (LVO) strokes. All other patients are transported to standard stroke-ready hospitals, which treat both hemorrhagic strokes and non-LVO ischemic strokes. However, when an LVO patient is initially sent to a hospital without thrombectomy capabilities, a secondary transfer is required—often introducing critical delays in care.

In the Madrid region, this secondary transfer process typically takes around two hours, factoring in time for initial evaluation, neuroimaging, and inter-hospital transport. Furthermore, internationally, the median time from symptom onset to arrival at the first hospital is between three and six hours (18), meaning that these additional delays could push patients beyond the recommended six-hour window for thrombectomy eligibility (19). Avoiding secondary transfers can therefore play a crucial role in ensuring timely treatment and improving patient outcomes.

While one theoretical strategy would be to route all suspected stroke patients directly to Stroke Units, this would likely overwhelm specialized resources, reducing availability for patients who truly need advanced care. Instead, a more balanced and efficient approach is to use predictive models for selective triage.

In our study, applying the LVO prediction model across the entire population resulted in 80% of ischemic LVO patients (Table 5) being correctly triaged and therefore could be correctly transferred to Stroke Units, with only 20% requiring secondary transfer. This represents a meaningful improvement over the current Madrid Direct prehospital scale, which has a 33% secondary transfer rate, implying a 13% relative reduction in unnecessary inter-hospital transfers and a more streamlined patient flow. Assuming that the 2,022 patient distribution is representative Figure 2, and that all LVO patients are eligible for thrombectomy—without considering exclusion criteria such as age or comorbidities—we estimate that approximately 86 patients per year in Madrid could benefit from faster, more direct access to definitive treatment. Although this analysis shows promising results, it is important to acknowledge the trade-off of increased false positives. Our model would result in 51% of extra patients being transported to Stroke Units, compared to 44% under the current scale, indicating a 7% increase in Stroke Unit patient load. Although this increase does not directly lead to secondary transfers, it may place additional pressure on limited specialized resources and potentially reduce the availability of timely care for true LVO cases.

**Figure 10:**
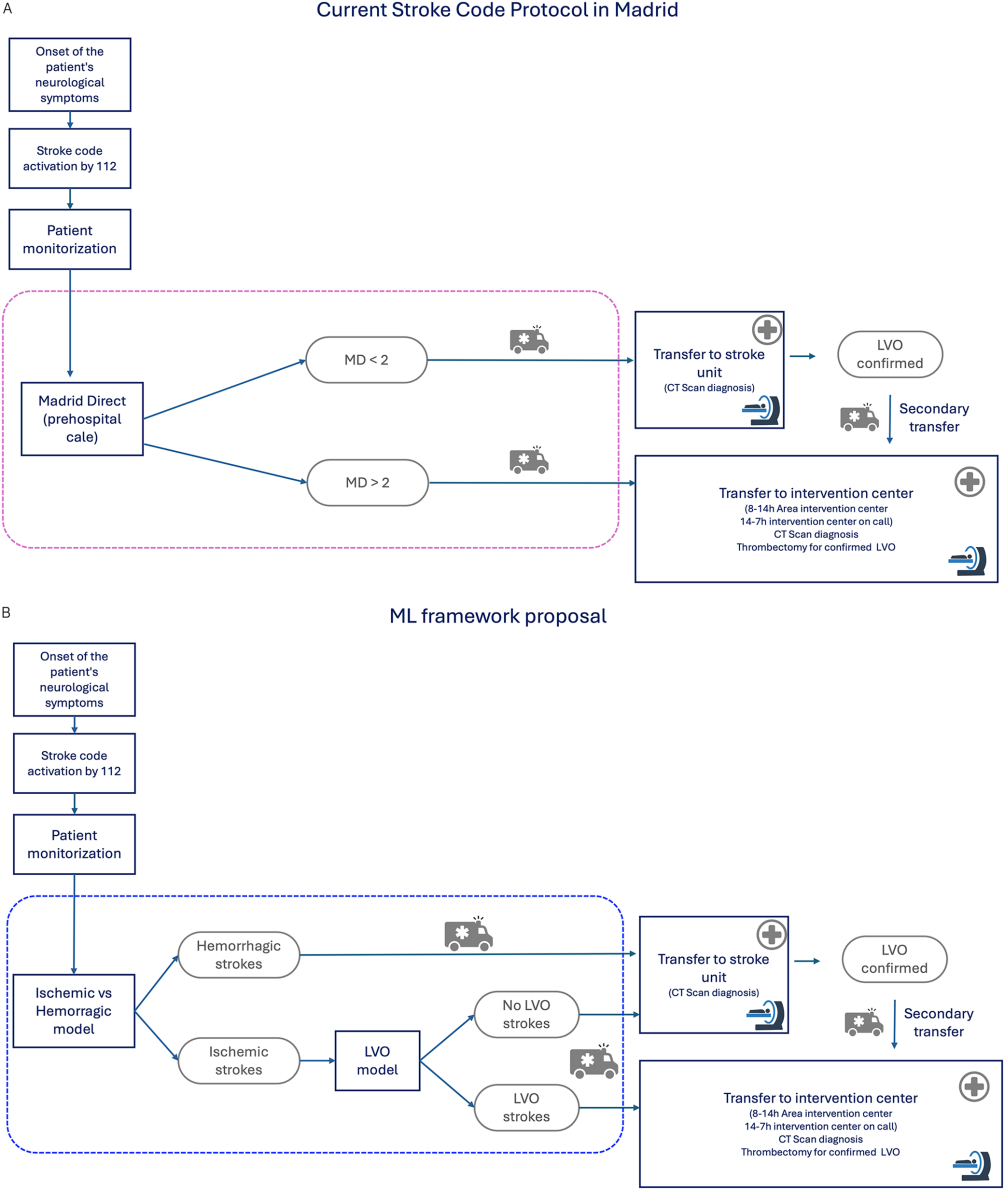
Proposed integration of machine learning (ML) models into the prehospital stroke triage workflow. The figure illustrates how ML-based decision support can be embedded within the existing Madrid Stroke Code protocol, similarly to how current clinical scales are used to guide hospital destination decisions. **(A)** Diagram showing current stroke code protocol. **(B)** Machine Learning framework proposal to adapt current stroke code protocol.

As a conclusion, this study shows the feasibility to develop and evaluate a machine learning (ML)-based framework designed to support prehospital stroke diagnosis. By leveraging data typically available in prehospital settings, the proposed system aims to enhance the accuracy of stroke identification, particularly in differentiating large vessel occlusion (LVO) cases, without disrupting established clinical protocols. This approach is designed to complement existing triage scales, such as those currently used in Madrid, which aim to identify patients who may benefit from direct transfer to comprehensive stroke centers. Rather than replacing the clinical judgment of emergency physicians, the proposed framework serves as a decision-support tool, intended to enhance diagnostic confidence and support more accurate patient routing. To illustrate this integration, Figure 10 presents how the current stroke code workflow could be augmented with ML model outputs, following a structure similar to that of the existing Madrid Stroke Code protocol (3). While this study successfully proves that machine learning models can help on decision making, still further work is required to obtain a practical software tool that can be used in the clinical scenario.

### 4.2 Limitations

A key limitation of the proposed pipeline stems from the reduced performance of the hemorrhagic model, which is largely attributed to the limited number of hemorrhagic stroke episodes in the dataset. Hemorrhagic strokes account for approximately 20% of all stroke cases, making them a minority class in this context. This low prevalence results in an imbalanced dataset, which significantly affects the model’s ability to generalize and detect hemorrhagic cases effectively. While techniques such as synthetic data generation might seem like a potential solution, they can introduce biases or artifacts that risk corrupting the model’s learning process—especially in critical applications where high generalization capacity is essential.

Addressing this limitation requires increasing the number of real hemorrhagic cases in the dataset. However, collecting a sufficiently large and representative sample poses a considerable challenge. It necessitates close coordination between emergency medical services and hospital-based stroke care units, ensuring consistent data capture across different stages of patient care. This level of integration is logistically complex and resource-intensive, but it is crucial to improve model performance and ensure reliability in real-world clinical settings. A larger and more balanced dataset would enable the development of more robust models capable of accurately identifying both ischemic and hemorrhagic strokes, ultimately contributing to faster and more effective clinical decision-making.

### 4.3 Future work

Future efforts will prioritize expanding and enhancing the dataset, with particular emphasis on increasing the number of hemorrhagic stroke cases. A more balanced representation of ischemic and hemorrhagic cases will improve the training process and potentially reduce false positives—especially for rarer stroke types. We are currently collecting data from 2023, which will allow for an assessment of the models’ temporal robustness. Given that the current models were trained on 2,022 data, it is crucial to evaluate whether shifts in clinical or demographic patterns—such as the preliminary observation of rising stroke incidence and a trend toward younger patients—impact model performance and generalizability. Moreover, adherence to a strict data collection protocol has improved over time, suggesting that the new dataset will not only be larger but also of higher quality.

Building on this improved dataset, future work will also explore strategies to enhance model robustness and flexibility. This includes integrating additional physiological signals and novel data sources that may enrich the feature space. Advanced training techniques—such as data augmentation, cost-sensitive learning, or synthetic oversampling—will be investigated to better handle class imbalances, ensuring reliable performance across stroke subtypes.

In parallel, efforts will continue toward real-world deployment, with a focus on ensuring technical feasibility and clinical usability in operational EMS settings. The models have been specifically selected to run on Panasonic Toughbook CF-H2 tablets used by SUMMA 112 personnel, operating on Windows 10 and integrated with SITREM, the regional EMS case management and EHR system. Each model remains computationally lightweight (14 KB for k-NN, 17 KB for Decision Tree, and 750 KB for Gradient Boosting), and a dedicated standalone application will be developed to encapsulate the entire inference pipeline—from variable transformation to prediction—functioning entirely offline to ensure reliability in low-connectivity environments.

A user-centered visual interface will be developed to display model predictions alongside confidence scores in an intuitive dashboard. This interface will support, but not override, clinical decision-making in the field. Its role is to enhance physician judgment by providing timely, data-driven insights at the point of care, with clear boundaries ensuring that final decisions remain in the hands of attending medical personnel.

To evaluate real-world performance, the application will be piloted in a single ambulance with oversight from one of the study physicians, pending ethics committee approval. This pilot deployment will allow for real-time validation of model predictions based on continuously monitored physiological parameters. Feedback from this phase will inform iterative improvements in both model behavior and interface usability. Depending on pilot outcomes, we aim to progressively scale implementation across the SUMMA 112 fleet in Madrid.

If clinical value is confirmed, updates to the Madrid Direct protocol will be proposed and reviewed by the Foro Ictus of the Comunidad de Madrid for potential official adoption. Additionally, to promote model generalizability and ensure adaptability across different EMS environments, we are pursuing collaborations with emergency services in Portugal and Seville (Spain). These partnerships aim to validate the models in diverse operational contexts and guide the development of a monitor-agnostic, scalable architecture informed by real-world data.

Lastly, future work will also explore ways to optimize interaction among multiple models—such that the output of one algorithm informs the next in a cohesive diagnostic chain. Careful coordination between models may reduce compounding errors and further enhance overall diagnostic accuracy. This integrative approach brings us closer to the practical implementation of machine learning–based stroke detection and triage in routine emergency care.

## Data Availability

The datasets presented in this article are not readily available because these data requires the permission of the La Princesa Hospital Ethics Committee to be used. Requests to access the datasets should be directed to mrios09@ucm.es. However, synthetic data that mimic the structure and characteristics of the original datasets are included in this publication to provide an illustrative example of the data and facilitate understanding of the methodology. Data can be found in Supplementary Material 3. Feature Selection and Model Pipeline scripts generated during this study are publicly available at https://github.com/Mariaridel/INDIA_Frontiers.

## Group member of SUMMA 112 India Research Group

The full list of contributing researchers is provided in Table S1 of the Supplementary Material 2.
