# Correction: Early stroke detection through machine learning in the prehospital setting

**DOI:** 10.3389/fcvm.2025.1708205

**Published:** 2025-10-23

**Authors:** María Ríos Delgado, Gemma Reig Roselló, Nicolas Riera-Lopez, José A. Vivancos, José L. Ayala

**Affiliations:** ^1^ Department of Computer Arquitecture and Automation, Universidad Complutense de Madrid, Madrid, Spain; ^2^ Servicio de Neurología, Hospital Universitario de La Princesa, IIS-Princesa – Instituto de Investigación Sanitaria Hospital Universitario de La Princesa, Madrid, Spain; ^3^ Stroke Commission, Madrid Emergency Medical Service (SUMMA 112), Madrid, Spain

**Keywords:** stroke, LVO, emeregency medical services, prehospital, machine learning, genetic algorithms, clinical data, hemodynamic data

An incorrect number was provided for Instituto de Salud Carlos III (ISCIII). The correct number is PI22/01454.

The original version of this article has been updated.

