# Supplementary material for: Early stroke detection through machine learning in the prehospital setting
Source: Front Cardiovasc Med. 2025 Aug 7;12:1629853. doi: 10.3389/fcvm.2025.1629853 (PMC12367740; doi:10.3389/fcvm.2025.1629853)
Supplement: Supplementary file 2 [file Datasheet1.pdf]

Supplementary Material

1 SUPPLEMENTARY TABLES

1.1 Tables

Table S1: Delfos Data Source Description. N: Numerical; DC: Dichotomous categorical; PC: Polytomous categorical; STR: String; DT: Date

Feature	Feature meaning	Category	Scale	Missing values (%)
GUID IA	Unique and hashed incident identifier	STR	Text	0
PCO	PCO file identifier	STR	Text	39.51
SEX	Gender of the patient	PC	M, H, D	0
DATE OF BIRTH	Date of birth	DT	Date	5.66
AGE	Age of the patient	N	Numerical	0
AGE TYPE	Type of age	PC	Years, Dayes	0
ID NUMBER	Hashed national ID number	STR	Text	0
HEALTH INSURANCE CODE	Patient ID hashed	STR	Text	0
INCIDENT ID	Hashed incident ID	STR	Text	0
CONSULTATION REASON	Reason for consultation	STR	Text	0
CAUSE	Cause of incident	PC	Medical, Accident, Other	0
INCIDENT DATE	Date of incident	DT	Date	0
ALLERGIES	Allergies	PC	Yes, No, Unknown	6.26
ALLERGY DESC	Description of allergies	STR	Text	84.53
MEDICAL HISTORY	Past medical history	PC	Yes, No, Unkown	2.57
MEDICAL HISTORY DESC	Description of past medical history	STR	Text	8.59
TREATMENT	Treatment administered	PC	Medication, Surgery, Other	5.46
TREATMENT DESC	Description of treatment	STR	Text	16.94
CONSULTATION REASON 2	Second reason for consultation	PC	Emergency, Routine	8.55
CURRENT CONDITION	Current illness	STR	Text	5.58
SECRETION ASPIRATION	Secretions aspiration	DC	Yes, No	1.24
TET	Endotracheal tube	DC	Yes, No	1.24

Feature	Feature meaning	Category	Scale	Missing values (%)
TET NUMBER	Number of endotracheal tubes	N	Numerical	98.39
CPAP	CPAP usage	DC	Yes, No	1.24
CPAP NUMBER	Number of CPAP usages	N	Numerical	100
MASK FIO2	O2 mask usage	DC	Yes, No	1.24
MASK FLOW	Flow mask usage	DC	Yes, No	94.09
MASK O2	O2 mask type	PC	Simple, Non-rebreather	95.02
OROPHARYNGEAL CANNULA	Oropharyngeal cannula usage	DC	Yes, No	1.24
CRICOTHYROTOMY	Cricothyrotomy performed	DC	Yes, No	1.24
RESUSCITATION BALLOON	Resuscitation bag usage	DC	Yes, No	1.24
VENTILATOR	Ventilator usage	DC	Yes, No	1.24
VENTILATOR MODE	Ventilator mode	PC	Assist, Control, Other	100
VENTILATOR P MAX	Max pressure on ventilator	N	Numerical	98.27
VENTILATOR FREQUENCY	Ventilator frequency	N	Numerical	98.23
AIR MIX	Air mix setting	N	Numerical	100
PEEP	Positive end-expiratory pressure	N	Numerical	98.39
TI TE	Inspiratory time	N	Numerical	98.39
VT	Tidal volume	N	Numerical	98.39
CARDIAC COMPRESSOR	CPR device used	DC	Yes, No	1.24
PACEMAKER	Pacemaker used	DC	Yes, No	1.24
PACEMAKER FREQUENCY	Pacemaker frequency	N	Numerical	100
PACEMAKER INTENSITY	Pacemaker intensity	N	Numerical	100
VENOUS LINES	Venous access	PC	Peripheral, Central	1.24
PERIPHERAL VEIN	Peripheral venous access	DC	Yes, No	1.24
CENTRAL VEIN	Central venous access	DC	Yes, No	1.24
INTRAOSSEOUS VEIN	Intraosseous access	DC	Yes, No	1.24
PERIPHERAL VEIN UPPER LEFT	Right peripheral venous access	DC	Yes, No	1.24
PERIPHERAL VEIN UPPER RIGHT	Left peripheral venous access	DC	Yes, No	1.24
PERIPHERAL VEIN LOWER LEFT	Right middle peripheral access	DC	Yes, No	1.24
PERIPHERAL VEIN LOWER RIGHT	Left middle peripheral access	DC	Yes, No	1.24
NECK VEIN	Neck access	DC	Yes, No	1.24

Feature	Feature meaning	Category	Scale	Missing values (%)
RIGHT INTERNAL JUGULAR VEIN	Internal jugular access	DC	Yes, No	1.24
SUBCLAVIAN VEIN	Subclavian access	DC	Yes, No	1.24
FEMORAL VEIN	Femoral access	DC	Yes, No	1.24
INTRAOSSEOUS VEIN UPPER LEFT	Right intraosseous access	DC	Yes, No	1.24
INTRAOSSEOUS VEIN UPPER RIGHT	Left intraosseous access	DC	Yes, No	1.24
INTRAOSSEOUS VEIN LOWER LEFT	Right middle intraosseous access	DC	Yes, No	1.24
INTRAOSSEOUS VEIN LOWER RIGHT	Left middle intraosseous access	DC	Yes, No	1.24
MEDICAL NOTES	Medical observations	STR	Text	36.18
NURSING NOTES	Nursing observations	STR	Text	18.71
EVOLUTION NOTES	Evolution observations	STR	Text	42.57
AIRWAY	Airway management	DC	Yes, No	6.30
VENTILATION	Ventilation status	DC	Yes, No	6.50
CAPILLARY REFILL	Capillary refill time	N	Numerical	17.31
PULSE	Pulse rate	N	Numerical	16.14
SKIN	Skin condition	PC	Normal, Abnormal	44.93
HEART AUSCULTATION	Cardiac auscultation findings	STR	Text	22.23
RIGHT EYE	Right eye condition	PC	Normal, Abnormal	22.97
LEFT EYE	Left eye condition	PC	Normal, Abnormal	23.05
GLASGOW EYE	Glasgow coma score (O)	N	Numerical	12.65
GLASGOW MOTOR	Glasgow coma score (M)	N	Numerical	12.85
GLASGOW VERBAL	Glasgow coma score (V)	N	Numerical	12.93
GLASGOW TOTAL	Total Glasgow coma score	N	Numerical	10.84
CONSCIOUSNESS LEVEL	Level of consciousness	PC	Alert, Drowsy, Unresponsive	14.53
STRENGTH UPPER RIGHT	Right upper limb strength	N	Numerical	10.84
STRENGTH UPPER LEFT	Left upper limb strength	N	Numerical	10.84
STRENGTH LOWER RIGHT	Right lower limb strength	N	Numerical	10.84

Feature	Feature meaning	Category	Scale	Missing values (%)
STRENGTH LOWER LEFT	Left lower limb strength	N	Numerical	10.84
SENSITIVITY UPPER RIGHT	Right upper limb sensation	N	Numerical	10.84
SENSITIVITY UPPER LEFT	Left upper limb sensation	N	Numerical	10.84
SENSITIVITY LOWER RIGHT	Right lower limb sensation	N	Numerical	10.84
SENSITIVITY LOWER LEFT	Left lower limb sensation	N	Numerical	10.84
ASSESSMENT COMMENTS	Evaluation comments	STR	Text	42.48
LUNG AUSCULTATION	Pulmonary auscultation findings	STR	Text	24.41
CRANIAL NERVES	Cranial nerves status	STR	Text	2.08
NEUROLOGICAL FOCALITY	Neurological focal findings	STR	Text	2.08
OCULOMOTOR REFLEXES	Oculomotor reflexes	DC	Yes, No	2.08
MUSCLE TENDON REFLEXES	Muscle-tendon reflexes	DC	Yes, No	2.08
FINGER NOSE	Nasal reflex	DC	Yes, No	2.08
ROMBERG	Romberg test	DC	Yes, No	2.08
MENINGEAL SIGNS	Meningeal signs	DC	Yes, No	2.08
NECK RIGIDITY	Neck stiffness	DC	Yes, No	2.08
BRUDZINSKI	Brudzinski sign	DC	Yes, No	2.08
KERNIG	Kernig sign	DC	Yes, No	2.08
ORIENTATION	Orientation status	PC	Oriented, Disoriented	2.08
TIME	Time of incident	DT	Date	2.08
PERSON	Person present	STR	Text	2.08
PLACE	Place of incident	STR	Text	2.08
AGITATED	Agitated state	DC	Yes, No	1.08
AGGRESSIVE	Aggressive behavior	DC	Yes, No	1.08
RETROGRADE AMNESIA	Retrograde amnesia	DC	Yes, No	1.08
CONSCIOUSNESS RECOVERED	Loss with recovery	DC	Yes, No	1.08
SPHINCTER RELAXATION	Interest relaxation	DC	Yes, No	1.08
BITING	Bite reflex	DC	Yes, No	1.08
BP SYSTOLIC	Blood pressure (systolic)	N	Numerical	56.42
BP DIASTOLIC	Blood pressure (diastolic)	N	Numerical	56.42
HR	Heart rate	N	Numerical	30.90
RR	Respiratory rate	N	Numerical	77.22
O2 SATURATION	Oxygen saturation	N	Numerical	44.53

Feature	Feature meaning	Category	Scale	Missing values (%)
ETCO2	End-tidal CO2	N	Numerical	99.59
GLUCOSE	Glucose level	N	Numerical	62.89
TEMPERATURE	Temperature	N	Numerical	70.24
GLASGOW EYE	Glasgow score (O)	N	Numerical	72.16
GLASGOW MOTOR	Glasgow score (M)	N	Numerical	72.40
GLASGOW VERBAL	Glasgow score (V)	N	Numerical	72.20
GLASGOW SCORE	Total GCS	N	Numerical	72.20
TRANSMISSION	Transmission type	PC	Telemetry, Manual	0
INTERVENTION	Intervention type	PC	Medical, Surgical	1.80
HOSPITAL ARRIVAL	Time of arrival at hospital	DT	Date	15.02
HOSPITAL	Name of hospital	STR	Text	4.13
COMPLETION	Completion status	PC	Completed, In progress	0
HOSPITAL ALERT	Hospital alert status	DC	Yes, No	0
ALERT TYPE	Type of alert	PC	Emergency, Non-emergency	0
RESOURCE	Resource used	STR	Text	0
RESOURCE TYPE	Type of resource	PC	Equipment, Personnel	0
ALERT DATE TIME	Alert date and time	DT	Date	0
ALERT COMMENTS	Alert comments	STR	Text	1
STROKE SYMPTOMS EVOLUTION HOURS	Time to evolution symptoms	N	Numerical	100
STROKE HIGH DEPENDENCY	Major dependence	DC	Yes, No	24.41
STROKE TERMINAL ILLNESS	Terminal illness	DC	Yes, No	24.41
STROKE OTHER EXCLUSION TEXT	Other exclusion text	STR	Text	98.43
STROKE SYMPTOMS START	Symptom onset time	DT	Date	100
STROKE OTHER EXCLUSION	Other exclusions	DC	Yes, No	24.41
STROKE INR	INR level	N	Numerical	99.07
STROKE NEUROLOGICAL FOCALITY	Neurological focal point	DC	Yes, No	24.41
STROKE GLUCOSE	Glucose level at stroke	DC	Yes, No	24.41
STROKE HR	Heart rate at stroke	DC	Yes, No	24.41
STROKE ECG	ECG results at stroke	DC	Yes, No	24.41
STROKE O2 SATURATION	Oxygen saturation at stroke	DC	Yes, No	24.41

Feature	Feature meaning	Category	Scale	Missing values (%)
STROKE RR	Respiratory rate at stroke	DC	Yes, No	24.41
STROKE TEMP	Temperature at stroke	DC	Yes, No	24.41
STROKE BP	Blood pressure at stroke	DC	Yes, No	24.41
STROKE PERIPHERAL VEINS	Peripheral access at stroke	DC	Yes, No	24.41
STROKE FACIAL PARALYSIS	Facial paralysis at stroke	DC	Yes, No	24.41
STROKE LIMB WEAKNESS	Limb paresis at stroke	DC	Yes, No	24.41
STROKE HEMIPARESIS	Hemiparesis at stroke	DC	Yes, No	24.41
STROKE MOTOR APHASIA	Motor aphasia at stroke	DC	Yes, No	24.41
STROKE ANOPSIAS	Anopsia at stroke	DC	Yes, No	24.41
STROKE HEADACHE	Headache at stroke	DC	Yes, No	24.41
STROKE ATAXIA	Ataxia at stroke	DC	Yes, No	24.41
STROKE OTHERS	Other symptoms at stroke	DC	Yes, No	24.41
STROKE ARM STRENGTH	Motor arm response at stroke	PC	0, 1, 2	24.41
STROKE LEG STRENGTH	Motor leg response at stroke	PC	0, 3, 4	24.41
STROKE ORDERS	Response to commands at stroke	PC	0, 7, 8	24.41
STROKE DEVIATION	Deviation at stroke	PC	0, 5, 6	24.41
STROKE DEFICIT	Deficit at stroke	PC	0, 9, 10	24.41
STROKE SYSTOLIC	Systolic pressure at stroke	PC	0, 11, 12, 13, 14, 15	24.41
STROKE SCORE	Score at stroke	N	Numerical	24.41
DRUG N	Sets of variables describing medication 1	STR	Text	0
TIME N	Sets of variables describing time of medication 1	DT	Date	0
ROUTE N	Sets of variables describing route of medication 1	STR	Text	0
DOSE N	Sets of variables describing dosage of medication 1	N	Numerical	0
UNIT N	Sets of variables describing unit of medication 1	STR	Text	0

Table S2: Hemodynamic Data Description. N: Numerical; DC: Dichotomous categorical; PC: Polytomous categorical; STR: String; DT: Date

Feature	Feature meaning	Category	Scale	Missing values (%)
patient id	Unique and hashed patient identifier	STR	Text	0
ECG evolution	Presence of more than 1 ECG to evaluate patient evolution	DC	True, False	0
Respiratory Rate Hz Median	Median respiratory rate in hertz	N	Numerical	42.42
Respiratory Rate Bpm Median	Median respiratory rate in breaths per minute	N	Numerical	42.42
Respiratory Rate Hz Min	Minimum respiratory rate in hertz	N	Numerical	42.42
Respiratory Rate Bpm Min	Minimum respiratory rate in breaths per minute	N	Numerical	42.42
Respiratory Rate Hz Max	Maximum respiratory rate in hertz	N	Numerical	42.42
Respiratory Rate Bpm Max	Maximum respiratory rate in breaths per minute	N	Numerical	42.42
NNI Counter Median	Median count of normal-to-normal (NN) intervals	N	Numerical	42.42
NNI Counter Min	Minimum count of NN intervals	N	Numerical	42.42
NNI Counter Max	Maximum count of NN intervals	N	Numerical	42.42
NNI Mean Median	Median of mean NN intervals	N	Numerical	42.42
NNI Mean Min	Minimum of mean NN intervals	N	Numerical	42.42
NNI Mean Max	Maximum of mean NN intervals	N	Numerical	42.42
NNI Min Median	Median of minimum NN intervals	N	Numerical	42.42
NNI Min Min	Minimum of minimum NN intervals	N	Numerical	42.42

Feature	Feature meaning	Category	Scale	Missing values (%)
NNI Min Max	Maximum of minimum NN intervals	N	Numerical	42.42
NNI Max Median	Median of maximum NN intervals	N	Numerical	42.42
NNI Max Min	Minimum of maximum NN intervals	N	Numerical	42.42
NNI Max Max	Maximum of maximum NN intervals	N	Numerical	42.42
HR Mean Median	Median of mean heart rate	N	Numerical	42.42
HR Mean Min	Minimum of mean heart rate	N	Numerical	42.42
HR Mean Max	Maximum of mean heart rate	N	Numerical	42.42
HR Min Median	Median of minimum heart rate	N	Numerical	42.42
HR Min Min	Minimum of minimum heart rate	N	Numerical	42.42
HR Min Max	Maximum of minimum heart rate	N	Numerical	42.42
HR Max Median	Median of maximum heart rate	N	Numerical	42.42
HR Max Min	Minimum of maximum heart rate	N	Numerical	42.42
HR Max Max	Maximum of maximum heart rate	N	Numerical	42.42
HR Std Median	Median of heart rate standard deviation	N	Numerical	42.42
HR Std Min	Minimum of heart rate standard deviation	N	Numerical	42.42
HR Std Max	Maximum of heart rate standard deviation	N	Numerical	42.42
NNI Diff Mean Median	Median of mean differences between successive NN intervals	N	Numerical	42.42

Feature	Feature meaning	Category	Scale	Missing values (%)
NNI Diff Mean Min	Minimum of mean NN interval differences	N	Numerical	42.42
NNI Diff Mean Max	Maximum of mean NN interval differences	N	Numerical	42.42
NNI Diff Min Median	Median of minimum NN interval differences	N	Numerical	42.42
NNI Diff Min Min	Minimum of minimum NN interval differences	N	Numerical	42.42
NNI Diff Min Max	Maximum of minimum NN interval differences	N	Numerical	42.42
NNI Diff Max Median	Median of maximum NN interval differences	N	Numerical	42.42
NNI Diff Max Min	Minimum of maximum NN interval differences	N	Numerical	42.42
NNI Diff Max Max	Maximum of maximum NN interval differences	N	Numerical	42.42
SDNN Median	Median of standard deviation of NN intervals	N	Numerical	42.42
SDNN Min	Minimum SDNN	N	Numerical	42.42
SDNN Max	Maximum SDNN	N	Numerical	42.42
RMSSD Median	Median root mean square of successive NN interval differences	N	Numerical	42.42
RMSSD Min	Minimum RMSSD	N	Numerical	42.42
RMSSD Max	Maximum RMSSD	N	Numerical	42.42
SDSD Median	Median of successive differences standard deviation	N	Numerical	42.42
SDSD Min	Minimum SDSD	N	Numerical	42.42
SDSD Max	Maximum SDSD	N	Numerical	42.42

Feature	Feature meaning	Category	Scale	Missing values (%)
NN50 Median	Median count of NN interval differences ¿50ms	N	Numerical	42.42
NN50 Min	Minimum NN50	N	Numerical	42.42
NN50 Max	Maximum NN50	N	Numerical	42.42
pNN50 Median	Median proportion of NN50	N	Numerical	42.42
pNN50 Min	Minimum pNN50	N	Numerical	42.42
pNN50 Max	Maximum pNN50	N	Numerical	42.42
NN20 Median	Median count of NN interval differences ¿20ms	N	Numerical	42.42
NN20 Min	Minimum NN20	N	Numerical	42.42
NN20 Max	Maximum NN20	N	Numerical	42.42
pNN20 Median	Median proportion of NN20	N	Numerical	42.42
pNN20 Min	Minimum pNN20	N	Numerical	42.42
pNN20 Max	Maximum pNN20	N	Numerical	42.42
Triangular Index Median	Median of HRV triangular index	N	Numerical	42.42
Triangular Index Min	Minimum triangular index	N	Numerical	42.42
Triangular Index Max	Maximum triangular index	N	Numerical	42.42
SD1 Median	Median of short-term variability (SD1)	N	Numerical	42.42
SD1 Min	Minimum SD1	N	Numerical	42.42
SD1 Max	Maximum SD1	N	Numerical	42.42
SD2 Median	Median of long-term variability (SD2)	N	Numerical	42.42
SD2 Min	Minimum SD2	N	Numerical	42.42
SD2 Max	Maximum SD2	N	Numerical	42.42
SD Ratio Median	Median SD1 to SD2 ratio	N	Numerical	42.42
SD Ratio Min	Minimum SD ratio	N	Numerical	42.42
SD Ratio Max	Maximum SD ratio	N	Numerical	42.42
Ellipse Area Median	Median area of the Poincaré ellipse	N	Numerical	42.42
Ellipse Area Min	Minimum ellipse area	N	Numerical	42.42
Ellipse Area Max	Maximum ellipse area	N	Numerical	42.42

Feature	Feature meaning	Category	Scale	Missing values (%)
Sample Entropy Median	Median sample entropy	N	Numerical	42.42
Sample Entropy Min	Minimum sample entropy	N	Numerical	42.42
Sample Entropy Max	Maximum sample entropy	N	Numerical	42.42
FFT Peak VLF Median	Median peak frequency in very low frequency band	N	Numerical	42.42
FFT Peak VLF Min	Minimum peak in VLF	N	Numerical	42.42
FFT Peak VLF Max	Maximum peak in VLF	N	Numerical	42.42
FFT Peak LF Median	Median peak frequency in low frequency band	N	Numerical	42.42
FFT Peak LF Min	Minimum peak in LF	N	Numerical	42.42
FFT Peak LF Max	Maximum peak in LF	N	Numerical	42.42
FFT Peak HF Median	Median peak frequency in high frequency band	N	Numerical	42.42
FFT Peak HF Min	Minimum peak in HF	N	Numerical	42.42
FFT Peak HF Max	Maximum peak in HF	N	Numerical	42.42
FFT Absolute VLF Median	Median absolute power in VLF	N	Numerical	42.42
FFT Absolute VLF Min	Minimum absolute VLF	N	Numerical	42.42
FFT Absolute VLF Max	Maximum absolute VLF	N	Numerical	42.42
FFT Absolute LF Median	Median absolute power in LF	N	Numerical	42.42
FFT Absolute LF Min	Minimum absolute LF	N	Numerical	42.42
FFT Absolute LF Max	Maximum absolute LF	N	Numerical	42.42
FFT Absolute HF Median	Median absolute power in HF	N	Numerical	42.42
FFT Absolute HF Min	Minimum absolute HF	N	Numerical	42.42
FFT Absolute HF Max	Maximum absolute HF	N	Numerical	42.42
FFT Relative VLF Median	Median relative power in VLF	N	Numerical	42.42
FFT Relative VLF Min	Minimum relative VLF	N	Numerical	42.42
FFT Relative VLF Max	Maximum relative VLF	N	Numerical	42.42

Feature	Feature meaning	Category	Scale	Missing values (%)
FFT Relative LF Median	Median relative power in LF	N	Numerical	42.42
FFT Relative LF Min	Minimum relative LF	N	Numerical	42.42
FFT Relative LF Max	Maximum relative LF	N	Numerical	42.42
FFT Relative HF Median	Median relative power in HF	N	Numerical	42.42
FFT Relative HF Min	Minimum relative HF	N	Numerical	42.42
FFT Relative HF Max	Maximum relative HF	N	Numerical	42.42
FFT Ratio Median	Median LF/HF power ratio	N	Numerical	42.42
FFT Ratio Min	Minimum LF/HF ratio	N	Numerical	42.42
FFT Ratio Max	Maximum LF/HF ratio	N	Numerical	42.42
FFT Total Power Median	Median total power across frequency bands	N	Numerical	42.42
FFT Total Power Min	Minimum total power	N	Numerical	42.42
FFT Total Power Max	Maximum total power	N	Numerical	42.42
Sinus Rhythm	Normal sinus rhythm	DC	True, False	0
Atrial Fibrillation	Atrial fibrillation	DC	True, False	0
Left Axis Deviation	Left axis deviation	DC	True, False	0
Sinus Tachycardia	Sinus tachycardia	DC	True, False	0
Prolonged QT Interval	Prolonged QT interval	DC	True, False	0
Poor R Wave Progression	Poor R-wave progression – likely normal variant	DC	True, False	0
Possible Anterior MI	Possible anterior myocardial infarction – unknown onset	DC	True, False	0
Inferior/Lateral ST-T Abnormality	ST-T segment abnormalities in inferior/lateral leads possibly due to myocardial ischemia	DC	True, False	0
Inferior MI	Inferior myocardial infarction – unknown onset	DC	True, False	0

Feature	Feature meaning	Category	Scale	Missing values (%)
Right Bundle Branch Block and LAH	Right bundle branch block and left anterior hemiblock	DC	True, False	0
Sinus Arrhythmia	Sinus arrhythmia	DC	True, False	0
Right Bundle Branch Block	Right bundle branch block	DC	True, False	0
Lateral T Wave Abnormality	Nonspecific T wave abnormalities in lateral leads	DC	True, False	0
Pacemaker Rhythm	Pacemaker rhythm – no further analysis provided	DC	True, False	0
Left Ventricular Hypertrophy	Left ventricular hypertrophy	DC	True, False	0
Generalized ST-T Abnormality	Generalized ST-T abnormalities possibly due to myocardial ischemia	DC	True, False	0
AFib with Rapid Ventricular Response	Atrial fibrillation with rapid ventricular response	DC	True, False	0
Left Anterior Hemiblock	Left anterior hemiblock	DC	True, False	0
Intraventricular Conduction Defect	Intraventricular conduction defect	DC	True, False	0
ST Depression at J Point	Nonspecific ST depression at the J point	DC	True, False	0
rSr Pattern in V1	Pattern rSr’ in V1 – likely normal variant	DC	True, False	0
Possible Inferior MI	Possible inferior myocardial infarction – unknown onset	DC	True, False	0
Demand Pacemaker Stimulation	Demand cardiac pacing	DC	True, False	0
Sinus Rhythm with First-Degree AV Block	Sinus rhythm with first-degree AV block at the upper limit	DC	True, False	0
Right Axis Deviation	Right axis deviation	DC	True, False	0
II P Positive	Positive P wave in lead II	N	Numerical	7.68
II P Negative	Negative P wave in lead II	N	Numerical	7.68

Feature	Feature meaning	Category	Scale	Missing values (%)
II Q Amplitude	Q wave amplitude in lead II	N	Numerical	7.68
II Q Duration	Q wave duration in lead II	N	Numerical	7.68
II R Amplitude	R wave amplitude in lead II	N	Numerical	7.68
II R Duration	R wave duration in lead II	N	Numerical	7.68
II S Amplitude	S wave amplitude in lead II	N	Numerical	7.68
II S Duration	S wave duration in lead II	N	Numerical	7.68
II R Prime Amplitude	R’ (second R) wave amplitude in lead II	N	Numerical	7.68
II R Prime Duration	R’ wave duration in lead II	N	Numerical	7.68
II S Prime Amplitude	S’ (second S) wave amplitude in lead II	N	Numerical	7.68
II S Prime Duration	S’ wave duration in lead II	N	Numerical	7.68
II ST J	ST segment level at the J point in lead II	N	Numerical	7.68
II ST 80	ST segment level at 80 ms after the J point in lead II	N	Numerical	7.68
II ST Slope	Slope of the ST segment in lead II	N	Numerical	7.68
II T Positive	Positive T wave in lead II	N	Numerical	7.68

Table S3. Clinical histories data definition. N: Numerical; DC: Dichotomous categorical; PC: Polytomous categorical; STR: String; DT: Date

Feature	Feature meaning	Category	Scale	Missing Values (%)
CIPA	Patient ID hashed	STR	Hashed text	0
HOSPITAL	Hospital patient is transferred to	STR	Text	0.42
DATE	Date of incident	DT	Date	0
STROKE TYPE	Type of stroke	PC	Ischemic, Hemorrhagic, Other	4.09
LVO	Large vessel occlusion	DC	Yes, No	4.27
TROMBOLISIS	Trombolysis treatment	DC	Yes, No	4.27
THROMBECTOMY	Thrombectomy treatment	DC	Yes, No	4.27
EXITUS	Death of patient	DC	Yes, No	14.88
