# Supplementary material for: Early stroke detection through machine learning in the prehospital setting
Source: Front Cardiovasc Med. 2025 Aug 7;12:1629853. doi: 10.3389/fcvm.2025.1629853 (PMC12367740; doi:10.3389/fcvm.2025.1629853)
Supplement: Supplementary file 3 [file Datasheet2.pdf]

Supplementary Material 2

1 RESEARCHERS CONTRIBUTORS

Table of all the researchers who have registered and contributed to the data collection for the INDIA research project by the Emergency Medical Service of Madrid (SUMMA 112)

Name of researcher	Professional Title
Adela Jacobo Martínez	Registered Nurse
Adoracion Jimenez Calvo	Medical Doctor
Aída Boil Ramajo	Registered Nurse
Alberto Garcia Villadangos	Emergency Medical Technician
Alberto Sanchez Lopez	Emergency Medical Technician
Aldara Gutierrez Cabello	Registered Nurse
Alejandro Carbonnero Aguilar	Registered Nurse
Alejandro García García	Medical Doctor
Alejandro Játiva Güémez	Emergency Medical Technician
Alejandro Jimenez Garcia	Registered Nurse
Alfonso Dominguez Valiño	Emergency Medical Technician
Alfonso Infantes González	Emergency Medical Technician
Alfredo Carrillo Moya	Registered Nurse
Alicia Durán Pozo	Registered Nurse
Alicia Navarro Martinez	Registered Nurse
Alicia Yagüez Rico	Registered Nurse
Almudena Crespo Ruiz	Medical Doctor
Almudena Garcia Serrano	Registered Nurse
Almudena Perez Santamaría	Emergency Medical Technician
Álvaro Rivas Pastor	Emergency Medical Technician
Ana Isabel Checa Nieto	Registered Nurse
Ana Isabel de la Peña Alba	Registered Nurse
Ana Isabel Jiménez Pérez	Medical Doctor
Ana Isabel Urbano López	Medical Doctor
Ana Maria Cabrera Vizcaino	Registered Nurse
Ana Maria Perez Alonso	Medical Doctor
Ana Moreno Serrano	Registered Nurse
Ana Roldán	Medical Doctor
Ana Sarciada Palencia	Medical Doctor
Angel Bueno Heras	Registered Nurse
Ángeles Gómez Muñoz	Medical Doctor
Antonia Gema Marin Gil	Medical Doctor
Antonio Apolinar Dorrego	Emergency Medical Technician
Antonio Sánchez Ortega	Medical Doctor
Aranzazu Beitia Avila	Registered Nurse
Aránzazu Beitia Avila	Registered Nurse
Aránzazu Martin Diez Madroñero	Registered Nurse
Aranzazu Ruiz González	Emergency Medical Technician
Arturo González Sanchez	Emergency Medical Technician
Atef Kanaan Kanaan	Medical Doctor
Beatriz Alba Carmona	Registered Nurse
Beatriz Jiménez Sánchez-Carpio	Medical Doctor
Beatriz Merchan Sanchez	Medical Doctor

Name of researcher	Professional Title
Begoña Herrera Calvo	Medical Doctor
Belen Gamero Donis	Registered Nurse
Belén Muñoz Isabel	Registered Nurse
Belén Rodríguez Pérez	Medical Doctor
Berta García Sánchez	Registered Nurse
Borja Rios Lama	Emergency Medical Technician
Bruno Civil	Registered Nurse
Candela Fran Torres	Registered Nurse
Cañas Luque Leticia	Registered Nurse
Carlos Alonso Blas	Medical Doctor
Carlos Edwin Neyra Pérez	Medical Doctor
Carlos González Oviedo	Emergency Medical Technician
Carlos Otero Lopez	Emergency Medical Technician
Carmen Sánchez Manzano	Emergency Medical Technician
Carmen Sandoval Gomez	Registered Nurse
Carolina Rodriguez Cuñado	Registered Nurse
Cristina Arranz Jimeno	Medical Doctor
Cristina Barreiro Martínez	Medical Doctor
Cristina Diaz Gonzalez	Registered Nurse
Cristina Gómez Usabiaga	Medical Doctor
Cristina Horrillo García	Medical Doctor
Cristina María Cantón Ortiz	Medical Doctor
Daniel Hernández Navarro	Emergency Medical Technician
David Carlos Lorenzo Simón	Emergency Medical Technician
David Maya López	Registered Nurse
David Sotillo Sanz	Emergency Medical Technician
Elena Figueredo San José	Medical Doctor
Elena Isabel Figueredo San José	Medical Doctor
Emilio Díaz González de las Cuevas	Emergency Medical Technician
Emmanuel Pelayo Martínez González	Medical Doctor
Emmanuel Pelayo, Martínez González	Medical Doctor
Enrique Platas Gil	Medical Doctor
Enrique Ramón Ariza González	Medical Doctor
Estela Baratas Crespo	Registered Nurse
Ester Armela Sánchez-Crespo	Medical Doctor
Fátima García Castaño	Registered Nurse
Felipe Alfonso Jimenez Pedreño	Medical Doctor
Fernando Abad Esteban	Medical Doctor
Fernando de Lama Garcia	Emergency Medical Technician
Fernando Lombardía Recio	Emergency Medical Technician
Fernando Saavedra Sanchez	Registered Nurse
Francisca Camacho Velez	Registered Nurse
Francisca Pilar Camacho Velez	Registered Nurse
Francisco Casas Saboya	Registered Nurse
Francisco Gallardo Torres	Emergency Medical Technician
Francisco Javier Garcia Pinar	Emergency Medical Technician
Francisco Javier López González	Emergency Medical Technician
Francisco José López González	Registered Nurse
Francisco José Sánchez García	Medical Doctor
Gabriel Diaz Medina	Registered Nurse
Gabriel Jesús Martínez-Villaseñor de Medina	Medical Doctor
Gema García Pastor	Registered Nurse
Gorka de Mues Alonso	Emergency Medical Technician

Name of researcher	Professional Title
Guadalupe González Naranjo	Registered Nurse
Guillermo Aguilar Mendoza	Medical Doctor
Guillermo Lago Flores	Registered Nurse
Guissely Yuly Quispe Figueroa	Medical Doctor
Gustavo Rangel Carredano	Medical Doctor
Héctor Cossio Díaz	Medical Doctor
Héctor García Plata	Registered Nurse
Iliana Veitia Fernández	Medical Doctor
Inmaculada Benito Martínez	Registered Nurse
Isabel Canales Corcho	Medical Doctor
Isabel de la Peña Alba	Registered Nurse
Isabel Lara Arévalo de Pablos	Registered Nurse
Javier Diaz-Tendero Rodriguez	Registered Nurse
Javier Piña Espinosa	Emergency Medical Technician
Javier Postigo Sánchez	Registered Nurse
Javier Prat López	Emergency Medical Technician
Jesús Pascual Martinez	Registered Nurse
Joaquín López Ciércoles	Registered Nurse
Joaquín Martín Cumbres	Medical Doctor
Joaquina Guerrero Parro	Medical Doctor
Jorge Casado Barranco	Medical Doctor
Jorge David López Laguna	Emergency Medical Technician
Jorge Pardiño Dopico	Emergency Medical Technician
Jorge Pérez Fernández	Emergency Medical Technician
Jose Antonio Gonzalez Mingueza	Medical Doctor
Jose Antonio Hortelano Alonso	Registered Nurse
Jose Antonio Martínez Rodriguez	Emergency Medical Technician
José Carlos Rivero López	Registered Nurse
Jose Ignacio Cerrón Reina	Medical Doctor
José Javier Rubio González	Medical Doctor
Jose Mª Navalpotro Pascual	Medical Doctor
José Manuel González Anguiano	Registered Nurse
José María Arevalo La Calle	Registered Nurse
José María Elcano Villanueva	Medical Doctor
Jose Mellado León	Emergency Medical Technician
Jose Miguel Lafuente Durá	Registered Nurse
Juan Carlos Francés Rodríguez	Emergency Medical Technician
Juan Domingo Rodríguez Marcos	Emergency Medical Technician
Juan Francisco Bejarano Ramirez	Registered Nurse
Juan Francisco García Alonso	Medical Doctor
Juan José Amo Moreno	Emergency Medical Technician
Juan Manuel Alonso Niño	Emergency Medical Technician
Julia de la Figuera Bayón	Registered Nurse
Julio Merlin Mandado	Medical Doctor
Julio Sancho Morcillo	Emergency Medical Technician
Krosmar Yuri Campos Mendoza	Medical Doctor
Laura de Elera Tapia	Medical Doctor
Leticia Bartolomé Bercial	Registered Nurse
Libertad Garrote Mateos	Emergency Medical Technician
Libia Falcon Vergaray	Medical Doctor
Lidia Roales Nieto	Registered Nurse
Lorena Barea Gomez	Registered Nurse
Lucía Benavent López	Registered Nurse

Name of researcher	Professional Title
Luis Alberto Castiblanco	Medical Doctor
Luis Alberto García Villadangos	Emergency Medical Technician
Luis Pizarro Gómez	Emergency Medical Technician
Luis Seoane Juiz	Registered Nurse
Luis Sizenando Leao Martins	Medical Doctor
M . Eugenia Martin Maldonado	Medical Doctor
M. Pilar Sáez González	Emergency Medical Technician
Mª Carmen Álvarez López	Emergency Medical Technician
Manuel Delgado García	Medical Doctor
Mar Sastre Ibarretxe	Medical Doctor
Margarita Barón Romero	Medical Doctor
Margarita Isabel Mariscal Martín	Medical Doctor
Maria Belen Estepa Lobato	Registered Nurse
María Begoña Melero García	Medical Doctor
María Criptana Díaz-Parreño Quirós	Registered Nurse
María Cristina Sánchez Lapeña	Registered Nurse
María Del Camino Fernández Del Blanco	Medical Doctor
María Del Carmen Martínez Caballero	Medical Doctor
María Del Mar Rojo Parra	Registered Nurse
María Dolores Ayuso Olmos	Registered Nurse
Maria Fraile Lopez	Medical Doctor
María Isabel Sánchez Sáenz	Medical Doctor
María Jesús Ibarra Arroyo	Emergency Medical Technician
María José González Sanavia	Registered Nurse
Maria Jose Parra Orellana	Registered Nurse
María José Querencia Gómez	Medical Doctor
Maria Luisa Arroyo Arroyo	Medical Doctor
María Luisa Arroyo Arroyo	Medical Doctor
María Luisa Dominguez Perez	Registered Nurse
María Redondo Lozano	Medical Doctor
Maria Teresa Martin de Rosales Cabrera	Medical Doctor
María. Criptana Díaz-Parreño Quirós	Registered Nurse
Marina Del Pilar Lanchas Porras	Registered Nurse
Mercedes García Lázaro	Medical Doctor
Miguel Angel Carvajal Ortega	Emergency Medical Technician
Miguel Palomo Fernandez	Medical Doctor
Miguel Santiuste García	Registered Nurse
Miriam Uzuriaga Martín	Medical Doctor
Mónica Baiget Llompart	Medical Doctor
Monica Coll Hernandez	Medical Doctor
Monica Rodriguez Fernandez	Medical Doctor
Mónica Roldán Fernández	Emergency Medical Technician
Monica Sabuquillo Castrillo	Registered Nurse
Mónica Simon Pastor	Registered Nurse
Montserrat Tur Palau	Medical Doctor
Mria Begoña de Pablo Aguilera	Registered Nurse
Navid Behzadi Koochani	Medical Doctor
Nélida Bernabé Vicente	Registered Nurse
Nicolás García Galán	Emergency Medical Technician
Noelia Lucio Cobo	Registered Nurse
Noelia Rodrigo Saiz	Medical Doctor
Nuria Arroyo Reino	Medical Doctor
Olga Escorial Sanz	Medical Doctor

Name of researcher	Professional Title
Olga Martín Olalla	Registered Nurse
Oliver Alonso Jimeenez	Emergency Medical Technician
Óscar Carrillo Fernández	Registered Nurse
Óscar García Novo	Emergency Medical Technician
Pablo Blanco Rodriguez	Medical Doctor
Pablo Lluva Arce	Emergency Medical Technician
Paloma E. Tamame Martín	Medical Doctor
Patricia Blanco Hermo	Medical Doctor
Patricia Caño Hortoneda	Registered Nurse
Patricia Carrasco Marcos	Registered Nurse
Patricia Gonzalez Rico	Registered Nurse
Patricia Roces Iglesias	Medical Doctor
Paula Jimenez Briongos	Registered Nurse
Pedro Tenorio Cañamas	Medical Doctor
Pilar González Jarandilla	Medical Doctor
Pilar López Vadillo	Medical Doctor
Pilar Medina Díaz	Registered Nurse
Pilar Varela Garcia	Registered Nurse
Rafael Omar Garcia Rodriguez	Emergency Medical Technician
Raquel Abejón Martín	Registered Nurse
Raquel Ahijado Hormigos	Medical Doctor
Raquel Gimeno Martin	Medical Doctor
Raquel Lafuente	Registered Nurse
Raquel Moreno Sánchez	Registered Nurse
Raul Marina Hernandez	Emergency Medical Technician
Ricardo Lopez-Nuño Del Campo	Emergency Medical Technician
Rita Gonzalez Garcia	Registered Nurse
Roberto Moran Lopez	Emergency Medical Technician
Rocio Segura Hall	Registered Nurse
Rocío Segura Hall	Registered Nurse
Rosa María Calvo Cervantes	Registered Nurse
Ruth Libertad Gomez Bravo	Registered Nurse
Sacramento Barrajon Martín de la Sierra	Registered Nurse
Salua Khanafer Galiano	Medical Doctor
Salvador Espinosa Ramirez	Medical Doctor
Sandra Sanz Sáez	Registered Nurse
Santiago Cuenca Garrido	Registered Nurse
Sara Delgado Hernández	Medical Doctor
Sara Hernández Martínez	Registered Nurse
Sara Maria Díaz Castro	Registered Nurse
Sara Martin Jimenez	Emergency Medical Technician
Sara Ortega Garzon	Medical Doctor
Sergio Moreno Sanz.	Registered Nurse
Soledad Barranca Mirón	Registered Nurse
Soledad Martin Fresneda	Medical Doctor
Sonia Castro Fernández	Registered Nurse
Sonia Díaz Granado	Registered Nurse
Susana López Aguado	Registered Nurse
Susana Peñuela	Medical Doctor
Tatiana Alonso Pena	Registered Nurse
Teresa Maroto de Hoyos	Medical Doctor
Teresa Sierra Garcia	Registered Nurse
Vicente Perezgrueso Alonso	Emergency Medical Technician

Name of researcher	Professional Title
Yassin Serroukh Serroukh	Emergency Medical Technician
Yazmina Martinez Martin	Registered Nurse
Zaida Marín Fernandez	Emergency Medical Technician

Table S1: Researchers who contributed to the data collection
